# Supplementary material for: Mapping the evolution of fertility support policies in China: A content and instrumental analysis
Source: PLoS One. 2025 Oct 9;20(10):e0332137. doi: 10.1371/journal.pone.0332137 (PMC12510515; doi:10.1371/journal.pone.0332137)
Supplement: S1 Appendix — (ZIP) [file pone.0332137.s001.zip › S1 Appendix. 226 original policy documents/87-环境保护部关于印发《环境保护部机关人事工作办法(试行)》、《环境保护部派出机构人事工作办法(试行)》、《环境保护部直属事业单位人事工作办法(试行)》的通知(FBM-CLI-4-118413).docx]

环境保护部关于印发《环境保护部机关人事工作办法(试行)》、《环境保护部派出机构人事工作办法(试行)》、《环境保护部直属事业单位人事工作办法(试行)》的通知

发布部门： 环境保护部(已撤销) 机构沿革

发文字号：环发〔2009〕69号

发布日期：2009.06.15

实施日期：2009.06.15

时效性： 现行有效

效力级别： 部门规范性文件

法规类别： 人事管理

环境保护部关于印发《环境保护部机关人事工作办法（试行）》、《环境保护部派出机构人事工作办法（试行）》、《环境保护部直属事业单位人事工作办法（试行）》的通知

（环发〔2009〕69号）

机关各部门，各派出机构、直属单位：

按照从严治部的要求，为推进干部人事工作科学化、民主化、规范化，进一步落实干部群众对人事工作的知情权、参与权、选择权、监督权，结合部机关、派出机构和直属单位实际，我部研究制定了《环境保护部机关人事工作办法（试行）》、《环境保护部派出机构人事工作办法（试行）》、《环境保护部直属事业单位人事工作办法（试行）》，经部党组会议审议通过，现予印发。请遵照执行。

附件：1.《环境保护部机关人事工作办法（试行）》

2.《环境保护部派出机构人事工作办法（试行）》

3.《环境保护部直属事业单位人事工作办法（试行）》

二○○九年六月十五日

附件一：

环境保护部机关人事工作办法（试行）

目录

第一章总则

第二章考试录用

第三章考核

第四章职务任免与职务升降

第五章选拔任用

第六章奖惩

第七章培训

第八章调入（出）、交流回避

第九章工资福利

第十章辞职辞退

第十一章退休

第十二章出国（境）审批

第十三章申诉控告

第十四章报告事项

第十五章人事档案管理

第十六章纪律与监督

第十七章附则

第一章总则

第一条为认真贯彻执行党的干部路线、方针、政策和国家有关法律、法规，推进部机关人事工作科学化、民主化、制度化、规范化，建设一支高素质机关干部队伍，加快推进环境保护历史性转变，建设生态文明，根据《公务员法》、《党政领导干部选拔任用工作条例》（以下简称《干部任用条例》）和有关法律、法规及规章，结合我部实际，制定本办法。

第二条部机关人事工作以马克思列宁主义、毛泽东思想、邓小平理论和“三个代表”重要思想为指导，深入贯彻落实科学发展观，坚持党管干部原则，坚持德才兼备、以德为先原则，坚持民主、公开、竞争、择优原则，坚持民主集中制原则，坚持从严治部、依法办事原则。

第三条部机关司级干部由部党组管理；处级干部由部党组委托部人事部门管理；科级干部由部人事部门管理。

第四条本办法适用于部机关司级以下（含本级，下同）公务员管理工作。

第五条部机关的机构编制管理按照中央和部有关规定执行。

第二章考试录用

第六条部机关录用担任主任科员以下职务公务员，采取考试与考察相结合的方法进行。加大从基层和实际工作一线录用公务员，除部分特殊职位外，逐步做到基本上录用具有两年以上基层工作经历的人员。

第七条录用公务员按照下列程序进行：

（一）制定录用计划。部人事部门根据编制空缺情况，商有关部门提出年度公务员录用计划，经分管人事工作的部长批准后，报中央公务员主管部门审批。

（二）发布招考公告。部人事部门根据批准的公务员录用计划拟定招考公告，向社会统一发布。

（三）报名与资格审查。网上公开报名，部人事部门根据有关规定和招考公告的职位条件，组织进行资格审查。

（四）笔试。公共科目由中央公务员主管部门统一确定和组织进行。根据需要，可以增加环保专业科目的笔试。

（五）面试。面试由部人事部门组织进行。

（六）确定考察人选。按照中央公务员主管部门的规定，部人事部门根据报考者的考试成绩，按照由高到低的顺序确定考察人选，报分管人事工作的部长批准。

（七）体检。部人事部门会同机关服务中心组织考察人选到中央公务员主管部门指定的医疗机构进行体检。

（八）考察。部人事部门会同用人部门组织对考察人选进行报考资格复审和考察。

（九）审批。根据报考者的考试成绩、体检结果和考察情况，由部人事部门提出拟录用人员名单，报分管人事工作的部长审批。

（十）公示与备案。部人事部门将部机关拟录用人员名单按要求向社会公示，公示时间为七个工作日。公示期满，对没有问题或者反映问题不影响录用的，部人事部门将拟录用人员名单报中央公务员主管部门备案。

第八条新录用公务员实行一年试用期制度。试用期从正式报到之日起计算，期满经考核合格的，按有关规定及时确定职务和级别；考核不合格的，按有关规定取消录用资格，并报中央公务员主管部门备案。

第九条确定新录用公务员的职务与级别，由本人对在试用期间的德、能、勤、绩、廉情况进行总结，所在部门对其试用情况进行全面考核，领导班子集体研究提出拟任职务的意见，填写《新录用公务员任职定级审批表》，报部人事部门审批。

第十条新录用公务员试用期满，任命职务、确定级别，按照下列规定执行：

（一）直接从高等院校毕业生中录用的、没有工作经历的公务员：大学本科毕业生、获得双学士学位的大学本科毕业生（含学制为六年以上的大学本科毕业生）、研究生班毕业和未获得硕士学位的研究生，任命为科员，定为二十五级；获得硕士学位的研究生，任命为副主任科员，定为二十四级；获得博士学位的研究生，任命为主任科员，定为二十二级。

（二）其他新录用的公务员：原具有公务员身份的，可参考其原任职务与级别，比照部机关同等条件人员，确定职务与级别。其他具有工作经历的，可根据其资历和工龄，比照部机关同等条件人员，确定职务与级别。

新录用公务员任职时间从试用期满之日起计算。

第三章考核

第十一条公务员考核坚持客观公正、民主公开、注重实绩的原则，实行领导与群众相结合，平时与定期相结合，定性与定量相结合的方法，按照规定的权限、条件、标准和程序进行。

第十二条对公务员的考核，以其职位职责和所承担的工作任务为基本依据，全面考核德、能、勤、绩、廉，重点考核工作实绩。

德，是指思想政治素质和个人品德、职业道德、社会公德等方面的表现。

能，是指履行职责的业务素质和能力。

勤，是指责任心、工作态度、工作作风等方面的表现。

绩，是指完成工作的数量、质量和效率。

廉，是指廉洁自律等方面的表现。

第十三条部机关公务员考核分为平时考核和定期考核。定期考核以平时考核为基础。

平时考核重点考核公务员完成日常工作任务、阶段工作目标情况以及出勤情况，可以采取谈话、听取汇报、考勤等方式进行，由主管领导予以审核评价。

定期考核采取年度考核的方式，于每年年底或者翌年年初进行。

第十四条年度考核的结果分为优秀、称职、基本称职、不称职四个等次。

第十五条确定为优秀等次须具备下列条件：

（一）思想政治素质高；

（二）精通业务，工作能力强；

（三）工作责任心强，勤勉尽责，工作作风好；

（四）工作实绩突出；

（五）清正廉洁。

第十六条确定为称职等次须具备下列条件：

（一）思想政治素质较高；

（二）熟悉业务，工作能力较强；

（三）工作责任心强，工作积极，工作作风较好；

（四）能够完成本职工作；

（五）廉洁自律。

第十七条公务员具有下列情形之一的，应确定为基本称职等次：

（一）思想政治素质一般；

（二）履行职责的工作能力较弱；

（三）工作责任心一般，或工作作风方面存在明显不足；

（四）能基本完成本职工作，但完成工作的质量和效率不高，或在工作中有较大失误；

（五）能基本做到廉洁自律，但某些方面存在不足。

第十八条公务员具有下列情形之一的，应确定为不称职等次：

（一）思想政治素质较差；

（二）业务素质和工作能力不能适应工作要求；

（三）工作责任心或工作作风差；

（四）不能完成工作任务，或在工作中有严重失误、失职；

（五）存在不廉洁问题，且情形较为严重。

第十九条年度考核在部党组的领导下，由部人事部门统一组织，机关各部门具体实施。

第二十条机关各部门成立考核小组。考核小组由领导班子全体成员和综合处主要负责人组成，根据实际情况吸收其他公务员代表参加。

第二十一条年度考核按下列程序进行：

（一）下发通知，对年度考核工作进行部署。

（二）公务员按照职位职责和有关要求进行总结，撰写述职述廉报告，并填写《公务员年度考核登记表》。

司级正副职领导干部在本部门全体人员会议上述职述廉，部人事部门、机关党委、驻部监察局派人参加。司级正副职领导干部述职述廉后，在部门全体人员范围内进行民主测评，部人事部门负责汇总统计测评结果。

司级非领导职务和处级以下公务员述职述廉由各部门组织实施。

（三）主管领导在听取群众意见的基础上，根据被考核公务员平时考核情况和个人总结，写出评语，提出考核等次建议。

（四）确定考核等次。司级正副职领导干部的考核等次，由部党组确定。司级非领导职务和处级以下公务员的考核等次，由各部门考核小组确定，部人事部门审核。

被确定为优秀等次的人数，一般掌握在各部门参加考核总人数的百分之十五以内。

（五）对确定为优秀等次的公务员，在部机关、派出机构和直属单位范围内进行公示，时间为三至七个工作日。

（六） 将考核结果以书面形式通知被考核公务员，并由本人签署意见。

第二十二条下述公务员年度考核按以下规定办理：

（一）病、事假累计超过考核年度半年的人员，不进行考核。

（二）新录用人员在试用期内参加本部门年度考核，不定等次，只写评语，作为任职、定级的依据。

（三）调任或者交流轮岗的人员，由现工作部门进行考核并确定等次，其调任或者交流轮岗前的有关情况由原单位提供。

（四）到地方挂职锻炼或驻外已满半年且未结束的人员，由挂职单位或驻外机构考核并确定等次；未满半年或当年已结束的人员，参加部机关的年度考核。

（五）因公派出学习、培训的人员，参加部机关的年度考核，根据学习、培训情况确定等次。

（六）被立案调查或受党纪、政纪处分的人员，年度考核按有关规定办理。

第二十三条公务员如对本人考核结果有异议申请复核，应当自知道考核结果之日起三十日内向部人事部门提出书面申请。

无正当理由不参加年度考核的公务员，经教育后仍然拒绝参加的，可直接确定其考核结果为不称职。

第二十四条《公务员年度考核登记表》存入本人档案，考核结果作为调整干部职务、级别、工资以及奖惩、培训、辞退的依据。

第四章职务任免与职务升降

第二十五条部机关公务员的职务任免与职务升降，按照干部管理权限，依照法定的条件和程序进行。

第二十六条部机关公务员的职务任免与职务升降管理权限规定如下：

（一）机关司级干部由部党组讨论决定。

（二）机关处级干部由干部所在部门提出建议，征得分管部领导同意后，部人事部门组织考察并经部人事部门司务会研究提出意见，报分管人事工作的部长批准。

（三）机关科级干部由干部所在部门提出意见，部人事部门司务会研究决定。

第二十七条部机关公务员具有下列情形的，应予任职，并应当按照规定确定级别：

（一）新录用公务员试用期满经考核合格的；

（二）通过调任、公开选拔等方式进入部机关的；

（三）晋升或者降低职务的；

（四）交流轮岗、挂职锻炼的；

（五）免职后需要新任职务的；

（六）其他原因需要任职的。

第二十八条公务员不得在企业和其他营利性机构兼任职务。因工作需要在机关外兼职的须经组织批准，不得领取兼职报酬；在职司级公务员兼任社会团体职务，由部人事部门商有关部门提出意见，征得分管部领导同意，报部长审批。处级以下公务员兼任社会团体职务，由所在部门提出意见，报部人事部门审批。

第二十九条部机关公务员具有下列情形之一的，应当按照规定程序予以免职：

（一）晋升职务后需要免去原任职务的；

（二）降低职务的；

（三）交流轮岗的；

（四）辞职或者调出部机关的；

（五）非组织选派，离职学习期限超过一年的；

（六）退休的；

（七）其他原因需要免职的。

公务员被免职后，应当及时办理公务交接手续。

第三十条部机关公务员有下列情形之一，其职务即自行免除，可不再办理免职手续：

（一）受到刑事处罚或劳动教养的；

（二）受到撤职或开除处分的；

（三）被辞退的；

（四）法律、法规及有关章程规定的其他情形。

第三十一条公务员晋升应当具备《公务员法》、《干部任用条例》规定的资格：

（一）晋升正司级、正处级领导职务的，应当分别担任副司级、副处级领导职务两年以上；

（二）晋升副司级、副处级领导职务的，应当分别担任正处级领导职务、主任科员三年以上；

（三）晋升巡视员、副巡视员的，应当分别担任副司级、正处级职务五年以上；

（四）晋升调研员、副调研员的，应当分别担任副处级、主任科员四年以上；

（五）晋升主任科员、副主任科员的，应当分别担任副主任科员、科员三年以上；

（六）部领导秘书按照《中共中央办公厅印发＜关于领导干部机要秘书选拔任用的意见＞的通知》（中办发[2009]10号）执行。

晋升处级以上领导职务，应当具有五年以上工龄、两年以上基层工作经历，一般应当具有在下一级两个以上职位任职的经历。晋升司级领导职务的，一般还应当有下一级领导职务的任职经历。

第三十二条部机关公务员晋升职务，应当逐级晋升。

表现特别优秀或者工作特殊需要的，可以破格或者越级晋升职务。破格和越级晋升条件和程序按照有关规定执行。

第三十三条实行领导职务任职试用期制度。晋升司、处级领导职务的，试用期为一年。

试用期满后，司级干部正式任职由部人事部门考核提出意见，征得分管部领导同意，报部长审批。处级干部正式任职由干部所在部门考核提出意见，部人事部门审批。

经考核不胜任的，按干部管理权限，免去现任职务，一般按试用前原职级适当安排工作。

第三十四条在年度考核中被确定为不称职的公务员，以及不胜任或者因其他原因不适宜担任现职又不宜转任同级其他职务的公务员，应当按照规定程序予以降职。

第三十五条公务员降职，一般降低一个职务层次。

公务员被降职的，其级别超过新任职务对应的最高级别的，应当同时降至新任职务对应的最高级别。

第五章选拔任用

第三十六条选拔任用司处级干部，严格按照《干部任用条例》和有关法律、法规的规定，按照酝酿、民主推荐、民主测评、考察、讨论决定等程序进行。

根据工作需要，经部党组批准，可以在机关或在京单位通过竞争上岗的方式，也可以面向社会公开选拔，产生任职人选。竞争上岗和公开选拔按有关规定实施。

第三十七条机关各部门司级职务出现空缺需要调整补充，由部党组进行充分酝酿，其中正职由部党组书记与其他部领导酝酿，副职由部党组委托部人事部门负责人沟通听取部领导意见，确定调整补充方案。部人事部门根据酝酿方案组织实施。

机关各部门处级职务出现空缺需要调整补充，所在部门主要负责人在领导班子内部酝酿，并征得分管部领导同意后，与部人事部门沟通。部人事部门综合分析研究，提出建议方案，经部长同意后组织实施。

第三十八条提任司处级干部，必须经过民主推荐提出考察对象。民主推荐包括会议投票推荐和个别谈话推荐。民主推荐的结果在一年内有效。

提任司级干部，由部人事部门组织民主推荐。其中副司级领导职务、司级非领导职务由所在部门领导班子根据民主推荐结果，与部人事部门沟通，结合平时考核、年度考核和后备干部考核情况，班子集体研究并征得分管部领导同意后提出考察对象建议，写出现实表现材料报部人事部门。

提任处级干部，由所在部门组织民主推荐，并根据民主推荐结果，结合平时考核、年度考核等情况，经领导班子集体研究并征得分管部领导同意后，提出考察对象建议，写出干部现实表现材料报部人事部门。

第三十九条参加会议投票推荐人员范围为：

推荐司处级干部人选，一般在干部所在部门全体干部范围内进行。根据工作需要和实际情况，司级正职领导职务人选推荐，也可以扩大参加推荐人员范围或在部机关正处长以上干部和派出机构、直属单位副司级以上干部范围内进行。

第四十条司级干部人选会议投票推荐，由部人事部门组织；处级干部人选会议投票推荐，由各部门组织。会议投票推荐实际到会人数不得低于应到人数的百分之八十。

第四十一条第一次推荐因票数分散，未产生考察人选的，根据实际情况，经沟通后，可以差额提出考察对象初步人选名单，进行二次会议投票推荐。参加人员范围一般为：所在部门处级以上干部。

根据需要，可以组织考察对象初步人选进行自述。

第四十二条参加个别谈话推荐人员范围为：

推荐司处级干部人选，一般在人选所在部门司级干部、内设处（室）主要负责人范围内进行。

根据需要，可适当扩大个别谈话推荐和征求意见的范围。

第四十三条司级领导职务人选考察对象应当在机关、派出机构和直属单位范围内进行公示，时间为三至七个工作日。

第四十四条提任司处级干部，由部人事部门组建干部考察组，采取民主测评、个别谈话、与考察对象面谈、民主评议、查阅干部档案和相关材料等方式，全面了解考察对象的“德、能、勤、绩、廉”等情况，注重考察干部的思想素质、工作实绩和廉政情况。

第四十五条民主测评主要了解考察对象工作情况和现实表现，重点了解贯彻落实科学发展观的情况。

第四十六条民主测评按照“德、能、勤、绩、廉”等五个类别设置测评内容和评价要点，并设置“是否同意其任职”等征求意见栏目。

测评内容主要包括政治态度、大局意识、思想品质，政策水平、组织协调、业务能力，精神状态、工作作风，履行职责成效、解决复杂问题、基础工作，以及廉洁自律等方面情况。

测评项目评价意见分为好、较好、较差、差，总体评价意见分为优秀、称职、基本称职、不称职。

第四十七条对考察对象进行民主测评，一般在所在部门全体干部范围内进行。根据需要，可扩大民主测评的范围。拟任职人选，优秀、称职率应达三分之二以上，赞成票应过半数。民主测评基本称职、不称职票达到三分之一或不同意任职票达到二分之一的人员，一般不列为任职人选。

第四十八条考察谈话主要深入了解考察对象的历史表现和现实表现，侧重了解干部在重大问题、关键时刻、艰苦复杂环境和完成部重点工作任务等方面的表现，全面了解干部的德才素质、表现情况、发展潜力和心理素质等。

第四十九条考察谈话参加人员范围如下：

（一）司级正职领导职务、巡视员和由副巡视员转任司级副职领导职务人选，考察谈话范围为：考察对象所在部门司级干部、内设处（室）主要负责人。

（二）司级副职领导职务、副巡视员和处长人选，考察谈话范围为：考察对象所在部门司级干部、内设处（室）主要负责人及所在处（室）全体人员。

（三）副处级领导职务和处级非领导职务人选，考察谈话范围为：考察对象所在部门司级干部和所在处（室）全体人员。

根据需要，可扩大考察谈话和征求意见的范围。

第五十条部人事部门就干部思想政治素质、履行“一岗双责”、理论和业务学习以及党风廉政情况听取直属机关党委的意见；就干部廉政情况书面征求驻部纪检组、监察局的意见；对需要进行经济责任审计的，应当委托审计部门按照有关规定进行审计。

第五十一条部人事部门司务会议根据民主推荐、民主测评和考察情况提出任免意见，按干部管理权限和有关程序报批。

第五十二条拟任司级干部在机关、派出机构和直属单位范围内公示，拟任处长在部机关公示，拟任副处长和处级非领导职务在本部门公示。公示时间为七至十五个工作日。

第五十三条各部门主要负责人任职由部主要领导、分管部领导谈话；司级副职领导职务和司级非领导职务，由分管部领导和部人事部门谈话；处级领导干部任免由部人事部门或所在部门谈话。驻部纪检组、监察局与任职干部进行廉政谈话。部人事部门负责向本人反馈考察情况。

第六章奖惩

第五十四条公务员奖励坚持公开、公平、公正原则，坚持精神奖励与物质奖励相结合、以精神奖励为主的原则，及时奖励与定期奖励相结合，按照规定的条件、种类、标准、权限和程序进行。

第五十五条对公务员、公务员集体的奖励分为：嘉奖、记三等功、记二等功、记一等功、授予荣誉称号。

（一）对表现突出的，给予嘉奖；

（二）对做出较大贡献的，记三等功；

（三）对做出重大贡献的，记二等功；

（四）对做出杰出贡献的，记一等功；

（五）对功绩卓著的，授予“人民满意的公务员”、“人民满意的公务员集体”或者“模范公务员”、“模范公务员集体”等荣誉称号。

第五十六条奖励应当按照下列程序和权限进行：

（一）公务员、公务员集体做出显著成绩和贡献需要奖励的，由所在部门在征求群众意见的基础上提出奖励建议，经部人事部门审核后，在一定范围内进行公示，时间为七个工作日。

（二）给予公务员集体、司级领导职务公务员奖励，以及司级非领导职务、处级以下公务员记三等功以上奖励，报部党组研究决定；给予司级非领导职务、处级以下公务员嘉奖，报部长审批。

（三）结合年度考核结果给予公务员的奖励，按年度考核程序进行，不重复报批。

《公务员奖励审批表》存入本人档案；《公务员集体奖励审批表》存入部人事部门文书档案。

第五十七条对在本职工作中表现突出、有显著成绩和贡献的，应当给予奖励。给予嘉奖和记三等功，一般结合年度考核进行。年度考核被确定为优秀等次的，予以嘉奖；连续三年被确定为优秀等次的，记三等功。

对于因同一事由已获得上级机关奖励的，部机关不再重复奖励。

第五十八条对获得奖励的公务员、公务员集体，按规定颁发奖励证书；获得记三等功以上奖励的，同时对公务员颁发奖章，对公务员集体颁发奖牌。

对获得奖励的公务员、公务员集体，按照规定标准给予一次性奖金。其中由中央公务员主管部门授予“人民满意的公务员”、“模范公务员”等荣誉称号的公务员，按照有关规定享受省部级以上劳动模范和先进工作者待遇。

第五十九条公务员违反法律、法规、规章以及行政机关的决定和命令，应当承担纪律责任的，依据《行政机关公务员处分条例》及法律法规有关规定给予处分。

给予部机关公务员处分，应当坚持公正、公平和教育与惩处相结合的原则，应当与其违法违纪行为的性质、情节、危害程度相适应，应当事实清楚、证据确凿、定性准确、处理恰当、程序合法、手续完备。

第六十条处分的种类为：警告、记过、记大过、降级、撤职、开除。

处分的时间期限为：警告，六个月；记过，十二个月；记大过，十八个月；降级、撤职，二十四个月。

第六十一条公务员在受处分期间不得晋升职务和级别，其中，受记过、记大过、降级、撤职处分的，不得晋升工资档次；受撤职处分的，按照规定降低级别。

公务员受开除以外的处分，在受处分期间有悔改表现，并且没有再发生违纪行为的，处分期满后，应当解除处分。解除处分后，晋升工资档次、级别和职务不再受原处分的影响。但是，解除降级、撤职处分的，不视为恢复原级别、原职务。

公务员受开除处分的，自处分决定生效之日起，解除其与环境保护部的人事关系。

第六十二条给予公务员处分要依法依纪进行，由部人事部门与机关纪委、驻部监察局沟通，研究提出意见，按照干部管理权限和规定程序报批。

机关纪委、驻部监察局对违法违纪的公务员进行调查处理，依照有关规定和程序办理。

第六十三条处分决定和解除处分的决定以书面形式通知本人。

部人事部门应当按照管理权限，及时将处分决定或者解除处分决定报中央公务员主管部门备案。

第七章培训

第六十四条部机关公务员培训坚持理论联系实际、以人为本、全面发展、注重能力、学以致用、改革创新、注重实效、科学管理的原则，按照有关规定和要求，根据工作需要和公务员队伍状况，有计划、有针对性地开展培训。

第六十五条培训内容主要包括：政府现代管理的发展趋势、环境保护管理与科技的新成果，落实科学发展观、推进环保历史性转变，建设资源节约型、环境友好型社会和生态文明等环境保护最新理论成果、总体工作部署和要求。

第六十六条部人事部门会同机关各部门制定公务员培训规划。担任司处级领导职务的公务员每五年应当参加党校、行政学院、干部学院或部人事部门认可的其他培训机构累计三个月以上的培训，其他公务员参加脱产培训的时间一般每年累计不少于十二天。

第六十七条部机关公务员培训分为初任培训、任职培训、专门业务培训和在职培训。

（一）初任培训是对新录用公务员进行的培训，培训内容主要包括政治理论、依法行政、公务员法和公务员行为规范、机关工作方式方法等基本知识及入部教育，重点提高新录用公务员适应部机关工作的能力。初任培训由部人事部门统一组织，在试用期内完成，时间不少于十二天。

未参加初任培训或培训不合格的不予以任职定级。

（二）任职培训是按照新任职务的要求，对晋升司处级领导职务的公务员进行的培训，培训内容主要包括政治理论、领导科学、政策法规、廉政教育及所任职务相关业务知识等，重点提高其胜任领导工作的能力。任职培训应当在公务员任职前或任职后一年内进行。任职培训可与中组部干部调训相结合。

调入部机关任职以及在机关晋升为副处级以上非领导职务的公务员，依照前款规定参加任职培训。

没有参加任职培训或者培训考试、考核不合格的公务员，应及时进行补训。

（三）专门业务培训是根据专项工作需要，对公务员进行的专业知识和技能培训，重点提高公务员从事专门业务工作所需的知识、能力和工作方法。专门业务培训由部人事部门归口管理，有关业务部门具体组织实施。

（四）在职培训是对全体公务员进行的以更新知识、提高工作能力为目的的培训。在职培训由部人事部门会同机关各部门组织实施。

在职培训考试、考核不合格的公务员，年度考核不得确定为优秀等次。

（五）部人事部门根据工作需要，组织开展公务员境外培训工作。三十天以内的境外培训，由国际司商有关部门选派；超过三十天的境外培训，由部人事部门商有关部门选派。中央组织部、人力资源和社会保障部、国家公务员局、国家外国专家局组织的境外培训，由部人事部门选派。

第六十八条健全和落实组织调训制度，干部所在部门应当按计划完成调训任务，公务员必须服从组织调训。

鼓励公务员利用业余时间自主选择参加培训。鼓励公务员本着工作需要、学用一致的原则，利用业余时间参加有关学历教育和其他学习。公务员在职学习取得的学历、学位，须经部人事部门按有关规定确认。学习费用按部有关规定执行。

第六十九条部人事部门和有关部门应当建立和完善机关公务员培训档案，对公务员参加培训的种类、内容、时间和考试考核结果等情况进行登记。

公务员培训情况作为考核司级领导班子和公务员本人的重要内容，是公务员职务调整的重要依据之一。

第八章调入（出）、交流回避

第七十条因工作需要，在规定的编制限额和职数内，部机关可调入工作人员。调入人选必须符合国家法律、法规、规章规定的公务员基本条件和拟任职务所要求的资格条件。

第七十一条调入公务员按下列要求办理：

（一）调入司级干部，由部党组酝酿沟通提出人选，部人事部门组织对人选进行考察并提出意见，报部党组讨论决定。

（二）调入处级以下干部，一般由用人部门商部人事部门提出人选，征得分管部领导同意后，部人事部门对人选进行考察并提出意见，报部长审批。

（三）个人向组织推荐调入人选，必须负责地写出推荐材料并署名。

（四）确定拟调任部机关副处级以上干部人选，按有关规定在调出单位和部机关予以公示。

（五）通过公务员考试录用、军转干部接收、竞争上岗和公开选拔等方式进入部机关的人员，按有关规定程序办理调入手续，不重复报批。

第七十二条公务员调出部机关，需提出书面申请，并按下列程序办理：

（一）司级公务员由部人事部门提出意见，分管部领导审核，报部长同意后，提交部党组审批；

（二）处级以下公务员由所在部门提出意见，部人事部门审核，分管部领导同意后，报部长审批。

第七十三条经批准调出人员，由部人事部门通知有关部门。调出人员要做好公务交接，及时到办公厅、规财司、国际司、机关党委、驻部监察局、机关服务中心和本人所在部门签署意见，部人事部门开具行政、工资介绍信。

第七十四条公务员可以在部机关内部交流轮岗，也可以在部机关与派出机构、直属单位以及其他部门之间交流任职。

第七十五条根据工作需要，加大部机关公务员交流轮岗力度。司级公务员根据工作需要在部门之间交流轮岗，处级以下公务员一般在本部门不同处（室）之间交流轮岗，也可以根据工作需要在部门之间交流轮岗。

第七十六条公务员有下列情形之一的，一般应当进行交流轮岗：

（一）担任处级以上领导职务并在同一职位任职五年以上的；

（二）担任处级以上非领导职务并在人财物管理和项目审批等一些敏感特殊职位上任职满五年的；

（三）除特殊专业岗位外，处级以下公务员在同一处（室）连续工作满十年的；

（四）工作需要的；

（五）需要回避的，或者其他原因需要交流轮岗的。

虽符合本条第（一）、（二）、（三）款规定的条件，但离最高任职年龄不满五年的处级以上公务员，可不进行部门之间的交流轮岗（可区别不同情况对其工作进行调整）。

第七十七条司级公务员的交流轮岗，由部人事部门提出意见，按程序报部党组审批。处级公务员跨部门的交流轮岗，由部人事部门商有关部门提出意见，征得分管部领导同意后，报部长审批；处级公务员在本部门内交流轮岗的，由所在部门提出意见，征得分管部领导同意，部人事部门审核后，报部长审批。主任科员以下公务员跨部门的交流轮岗，由接收部门商所在部门提出意见，报人事司审批；在本部门内交流轮岗的，由所在部门研究决定，报部人事部门备案。

第七十八条公务员离任前应当办理公务交接手续，负责财务工作的司级公务员离任，按规定应当由有关部门进行离任审计。

第七十九条按照公务员管理有关法律、法规和规章的规定，实行回避制度。具有夫妻关系、直系血亲关系、三代以内旁系血亲以及近姻亲关系的，不得在同一机关担任双方直接隶属于同一领导人员的职务或者有直接上下级领导关系的职务，也不得在其中一方担任领导职务的机关从事组织、人事、纪检、监察、审计和财务工作。

公务员在任职或从事公务活动时，应当主动、如实报告应回避事宜。

第八十条干部交流轮岗、回避要坚持个人服从组织的原则，被确定为交流轮岗、回避的公务员必须服从组织决定，对无正当理由拒不服从组织决定的，要给予批评教育；坚持不改的，予以免职。

第八十一条部机关有计划地选派公务员援藏、援疆、扶贫和挂职锻炼。

挂职锻炼的司级公务员一般不超过五十岁；处级公务员一般不超过四十五岁。部自主安排的挂职锻炼，时间一般为一至二年；由中央有关部门统一组织的挂职锻炼，按规定的挂职锻炼时间执行。

第八十二条有计划组织安排没有基层工作经历的年轻公务员到基层学习锻炼。到基层学习锻炼的时间一般为一年。

第九章工资福利

第八十三条公务员执行国家规定的工资制度、工资标准和工资政策，执行国家规定的各项津贴、补贴标准。

第八十四条对年度考核称职以上的公务员，发给一次性奖金，奖金数额按国家规定执行。

第八十五条公务员实行正常晋升工资制度。

累计两年考核被确定为称职以上等次的，在所定级别对应工资标准内晋升一个工资档次。

累计五年考核被确定为称职以上等次的，在所任职务级别范围内晋升一个级别。

第八十六条新录用、调入、晋升职务、降职的公务员和军队转业干部，工资和津贴补贴按照国家有关规定进行核定。

第八十七条公务员工资变动按下列审批权限进行：

（一）司级公务员因职务变动，需调整职务工资和级别工资的，由部人事部门负责核定。有特殊情况的，报分管人事工作的部长审批；

（二）处级以下公务员因职务变动，需调整职务工资和级别工资的，由部人事部门负责审批；

（三）公务员正常晋升工资或国家统一调整工资时，由部人事部门制定调整工资方案，报分管人事工作的部长审批后组织实施。

第八十八条对生活特别困难的公务员给予的补助费，按国家有关规定确定。对援藏、援疆、扶贫和挂职锻炼的公务员给予一定的生活补助，补助标准按国家和部有关规定执行。

第八十九条实行年休假制度。工作年限满一年不满十年的，年休假五天；已满十年不满二十年的，年休假十天；已满二十年的，年休假十五天。当年参加工作的，不享受年休假。具体按我部《机关工作人员带薪年休假制度的实施意见》执行。

第九十条公务员夫妻双方两地分居一年以上，又不能在公休假日团聚的，在对方未享受探亲假的情况下，可以享受探望配偶的待遇。公务员的父母均不是北京居民且在京外工作或生活，不能在公休假日团聚的，可以按规定享受探望父母的待遇。

（一）公务员探望配偶的，每年给予探亲假一次，假期三十天。未婚公务员探望父母，原则上每年给予探亲假一次，假期二十天。已婚公务员探望父母的，每四年给予探亲假一次，假期二十天。根据实际需要，给予路程假。

（二）公务员探望配偶和父母的往返路费按有关规定报销。

（三）享受探亲假的公务员，由本人提出申请，参照年休假的程序进行审批备案。

第九十一条符合法定结婚年龄的公务员结婚可享受婚假，假期三天；晚婚（男满二十五周岁，女满二十三周岁）的增加奖励假七天。

第九十二条女性公务员生育享受九十天产假；经所在部门批准可再增加产假三个月，但减少三年独生子女奖励费；晚育（女满二十四周岁）女性公务员除享受九十天产假（不含剖腹产假）外，增加奖励假三十天，该奖励假也可以由男方享受；剖腹产假根据医院出具的证明办理。

第九十三条公务员因病休假，需持医生诊断证明，经本部门领导批准，可以休病假。

病假两个月之内，工资照发。病假超过两个月的，从第三个月起按下列标准发给工资：工作年限不满十年的，发给本人工资的百分之九十；工作年限满十年的，工资照发。病假累计超过六个月的，按下列标准发给工资：工作年限不满十年的，发给本人工资的百分之七十；工作年限满十年和十年以上的，发给本人工资的百分之八十。

第九十四条公务员的配偶、直系亲属、岳父母、公婆死亡后，可请三天（不含往返路程时间）的丧假。

第九十五条第八十九条至第九十四条所述假期，相互之间可独立享受。除年休假、婚假和丧假外，均包括双休日和法定假日。

第十章辞职辞退

第九十六条部机关公务员辞去公职，应当提出书面申请。公务员有下列情形之一的，不得提出辞去公职：

（一）未满最低服务年限的；

（二）在涉及国家秘密等特殊职位任职或者离开上述职位不满国家规定的脱密期限的；

（三）重要公务尚未处理完毕，须由本人继续处理的；

（四）正在接受纪检机关（监察部门）、司法机关调查或者审计机关审计的；

（五）国家规定的其他不得辞去公职的情形。

第九十七条公务员辞去公职按下列程序办理：

（一）本人提出辞职申请，填写《公务员辞职申请表》。

（二）所在部门提出意见，征得分管部领导同意后，按干部管理权限和规定程序办理。

（三）审批结果以书面形式通知申请辞职的公务员。

公务员申请辞去公职，未经批准的，不得擅自离职；擅自离职的，给予开除处分。

第九十八条新录用的公务员最低服务年限为五年（含试用期）。未满最低服务年限的，不得提出调离部机关或出国自费留学、辞去公职的申请。对擅自离职的，给予开除处分。

组织安排一年以上脱产培训或由组织选派到国（境）外培训、工作六个月以上的公务员，自回部工作之日起，最低服务年限为三年，在服务期内不得提出辞去公职或调出，擅自离职的，给予开除处分，并赔偿培训费用。

经组织决定调出的可不受本条规定的限制。

第九十九条担任领导职务的公务员，需要辞去领导职务的，按国家有关规定办理。

第一百条部机关公务员有下列情形之一的，予以辞退：

（一）在年度考核中，连续两年被确定为不称职的；

（二）不胜任现职工作，又不接受其他安排的；

（三）因机构调整、撤销、合并或者缩减人员编制需要调整工作，本人拒绝合理安排的；

（四）不履行公务员义务，不遵守公务员纪律，经教育仍无转变，不适合继续在机关工作，又不宜给予开除处分的；

（五）旷工或者因公外出、请假期满无正当理由逾期不归连续超过十五天，或者一年内累计超过三十天的。

第一百零一条对有下列情形之一的公务员，不得辞退：

（一）因公致残，被确认丧失或者部分丧失工作能力的；

（二）患病或者负伤，在规定的医疗期内的；

（三）女性公务员在孕期、产期、哺乳期内的；

（四）法律、法规规定的其他不得辞退的情形。

第一百零二条辞退公务员按下列程序办理：

（一）所在部门在核准事实的基础上，经部门领导班子集体研究提出建议，填写《辞退国家公务员审批表》，按管理权限报批。

（二）辞退司级公务员，由部党组审批；辞退处级以下公务员，由部人事部门提出审核意见，分管部领导同意后，报部长审批。

（三）辞退决定应当及时以书面形式通知被辞退的公务员。

第一百零三条公务员辞去公职或者被辞退，应当在接到通知之日起半个月内，办理公务交接以及相关手续，交还个人使用的公共财物，必要时应当接受财务审计。

第一百零四条公务员辞去公职或者被辞退，自批准之月的下月起停发工资。被辞退的公务员，按国家有关规定领取一定的辞退费。

第一百零五条公务员辞去公职或者被辞退，其人事档案按规定转至人才流动服务机构。

第十一章退休

第一百零六条除国家另有规定外，公务员符合下列条件之一的，应当退休：

（一）男年满六十周岁的；

（二）处级以下女干部年满五十五周岁，或者司级女干部年满六十周岁的；

（三）完全丧失工作能力的。

第一百零七条公务员符合下列条件之一的，本人提出要求，经批准可提前退休：

（一）工作年限满三十年的；

（二）离规定的退休年龄不足五年，且工作年限满二十年的；

（三）符合国家规定可以提前退休的其他情形的。

第一百零八条根据中央要求，凡到达退休年龄的公务员应当及时办理退休手续。司级公务员退休，由部人事部门提出意见，分管部领导审核后，报部长批准。

处级以下公务员退休，由所在部门提出意见，报部人事部门批准。

第一百零九条个别到达退休年龄确因工作需要继续留任，暂不办理退休手续的，应按管理权限进行审批，继续留任时间最长一般不超过一年。

第一百一十条退休费从批准退休之月的下月起发放，由部人事部门按有关规定进行核定，并填写《退休审批表》存入本人档案。

退休的公务员福利待遇按国家有关规定执行。

第一百一十一条对已办理退休手续的人员，从办理退休手续之月的下月移交离退休干部机构管理。离退休干部机构要认真履行职责，做好离退休人员的管理和服务工作。

第十二章出国（境）审批

第一百一十二条出国任务（项目）审批，按部有关规定执行。公务员因公出国（境），所在部门负有出国人员审查的直接责任，部人事部门负有出国人员政治审查的责任。

（一）出国（境）公务员必须政治可靠、历史清楚、热爱祖国、身体健康、作风正派、遵守纪律。进部机关工作时间不满一年的公务员，原则上不派遣出国（境）执行公务。

（二）正处级以上公务员因公临时出国（境），由所在部门填写《因公出国（境）人员备案表》报部人事部门备案；其他公务员因公临时出国（境），在办理出国（境）任务批件后，由所在部门填写《因公出国（境）人员审查表》，经部人事部门审查，并由部党组授权部人事部门负责人审批。出国人员审查有效期为三年，有效期内再次出国的人员，不再办理因公出国（境）审查手续，但须填写《因公出国（境）人员备案表》报部人事部门备案。

（三）公派出国留学（进修、培训）的公务员，在国外的期限要严格执行批准的时间，一般不得延长；确需延期的，应当提前提出申请，按干部管理权限报批，未经批准逾期不归的，按有关规定处理。

第一百一十三条公务员配偶为外交人员，本人随任或探亲，按国家有关规定办理。

公务员中的归侨、侨眷申请出国（境）探望配偶、父母、兄弟姐妹的，公务员的配偶为留学年限在三年以上的公派出国研究生，或婚后在国（境）外学习期限达一年以上，申请出国 （境）探亲的，可予批准，假期按国家有关规定执行。

第一百一十四条公务员因私出国（境），按事假对待，填写《因私事出国（境）请假申请表》，并应当按照管理权限履行审批手续：

（一）司级正职请假，由本人提交书面请假报告和有关材料，经部人事部门审核，分管部领导同意后，报部长审批。

（二）司级副职及其他在职公务员请假，由本人向所在部门提交书面请假报告和有关材料，经所在部门签署意见，部人事部门审核后，报分管部领导审批。

（三）机关离退休的司级干部，由本人向离退休干部机构提交书面请假报告和有关材料，经离退休干部机构审核后，由部党组授权部人事部门领导审批。离退休处级以下人员，根据有关规定，不再办理审批手续，但应当事先向离退休干部机构报告。

第一百一十五条经批准因私出国（境）的公务员，在境外的时间按有关规定执行。出国（境）人员应当及时将本人离境日期报告所在部门和部人事部门，逾期不归者，按有关规定处理。

经批准因私出国（境），其费用（包括往返旅费、在境外医疗费等）自理。

第十三章申诉控告

第一百一十六条部机关公务员对涉及本人的下列人事处理不服，可以自被告知该人事处理之日起三十日内向部人事部门申请复核，对复核结果不服的可以自接到复核决定之日起十五日内向中央公务员主管部门提出申诉；也可以不经复核直接提出申诉：

（一）处分；

（二）辞退或者取消录用；

（三）降职；

（四）年度考核定为不称职；

（五）免职；

（六）申请辞职或提前退休未予批准；

（七）未按国家规定确定或者扣减工资、福利、保险待遇；

（八）法律、法规规定可以申诉的其他情形。

公务员对处分不服向行政监察机关申诉的，按照《行政监察法》的规定办理。

第一百一十七条公务员提出申诉，应当实事求是，不得捏造事实，诬告、陷害他人。

复核、申诉期间不停止人事处理的执行。

公务员不因申请复核、提出申诉而被加重处理。

第一百一十八条公务员对机关及其领导人员侵犯其合法权益的，可以依法向上级机关或者有关的专门机关提出控告。

第十四章报告事项

第一百一十九条根据中共中央办公厅《关于党员领导干部报告个人有关事项的规定》，副处级以上党员干部应当向组织报告下列事项：

（一）本人的婚姻变化情况；

（二）本人持有因私出国（境）证件的情况；

（三）本人因私出国（境）的情况；

（四）子女与外国人、港澳台居民通婚的情况；

（五）配偶、子女出国（境）定居及有关情况；

（六）配偶、共同生活的子女（指同财同居的子女，下同）私人在国（境）外经商办企业的情况；

（七）配偶、共同生活的子女担任外国公司驻华、港澳台公司驻境内分支机构主管人员的情况；

（八）配偶、子女被司法机关追究刑事责任的情况；

（九）本人认为应当报告的其他事项。

第一百二十条应当报告的有关事项，党员干部应当在事后三十日内填写《党员领导干部个人有关事项报告表》，向部人事部门报告。因特殊原因不能按时报告的，特殊原因消除后应当及时补报，并说明原因。报告人认为有需要请示的事项，可以向部人事部门书面请示。部人事部门应当认真研究，及时答复报告人。报告人应当按照组织答复意见办理。

部人事部门应当于每年三月一日前将上一年度党员领导干部报告有关事项的情况汇总后报部党组和驻部纪检组。

第一百二十一条不按规定报告、不如实报告、隐瞒不报或不按组织答复意见办理的，视情节轻重，采取批评教育、限期改正、责令做出检查、诫勉谈话、通报批评等方式处理。

第一百二十二条根据中共中央办公厅《关于党政机关县（处）级以上领导干部收入申报的规定》，副处级以上公务员应当填写《处以上领导干部收入申报表》。各部门负责接受本部门申报人的收入申报，并将申报材料报部人事部门备案。

第十五章人事档案管理

第一百二十三条部人事部门负责部机关公务员的人事档案管理工作。指定专人管理人事管理软件和公务员人事档案，及时做好公务员人事档案的审核、归档和整理工作，严禁无关人员进入档案室，严格归档程序，确保档案安全。

第一百二十四条公务员人事档案信息的归档按下列程序进行：

（一）公务员个人接受培训或学历证明等材料原件，由本人或所在部门及时送部人事部门审核后，归入本人档案。

（二）公务员的年度考核登记表、考察材料、《干部任免审批表》、工资变动审批（备案）表和奖惩审批表等材料，由部人事部门及时归入本人档案。

（三）公务员婚姻及其配偶、子女学习、工作等情况发生变化，本人应当在三十日内向所在部门和部人事部门报告，部人事部门核实后及时变更干部管理信息。

第一百二十五条管理人事档案，应建立健全并严格执行以下制度：

（一）人事档案材料收集制度。凡是应收入干部人事档案的材料均应按时间、按要求归入个人档案。

（二）人事档案材料鉴别归档制度。要按规定判断材料是否属归档范围，未经鉴别和批准，不得擅自销毁干部档案材料。

（三）人事档案查（借）阅制度。查（借）阅档案，必须经过批准，履行登记手续。任何组织不得批准任何人查（借）阅本人及本人公务回避对象的档案。严禁查（借）阅人在档案材料上涂改、划圈、增删、折叠或损坏档案材料，并不得泄露档案内容。未经同意，不得擅自摘抄、复制档案内容。

（四）人事档案传递制度。人事档案必须通过机要交通或派专人送取，严禁邮寄或交干部本人自带。传递档案要填写传递单，并催要回执。

（五）人事档案保管保密制度。部人事部门应按有关规定建立可靠的档案保管库房。严禁任何人私自保管他人档案。严格遵守保密规定，防止人事档案毁损、丢失、散落、失密、泄密等事故发生。

第十六章纪律与监督

第一百二十六条部人事部门按照有关规定，受理有关人事管理工作的举报和申请复核，制止、纠正违反本规定的行为，并对有关责任人做出处理或者提出处理意见。

第一百二十七条实行党政领导干部选拔任用工作责任追究制度。用人失察失误造成严重后果的，应当根据具体情况，追究主要责任人以及其他直接责任人的责任。

第一百二十八条对违反人事工作原则、程序、纪律的，对当事人进行批评教育，情节严重的，按照有关规定予以严肃处理。

第一百二十九条部人事部门应当在选人用人上切实为部党组当好参谋，落实群众对干部工作的知情权、参与权、选择权和监督权，自觉接受监督。

人事干部要坚持原则，公道正派，坚持讲党性、重品行、作表率，模范遵守党的干部人事工作纪律和各项规定，认真执行组工干部“十严禁”纪律要求，自觉抵制用人上的不正之风。

第十七章附则

第一百三十条部机关人事管理的现行规定与本办法不一致的，以本办法为准；国家对机关人事管理工作有新规定的，从其规定。

第一百三十一条本办法由部行政体制与人事司负责解释。

第一百三十二条本办法自发布之日起施行。

附件二：

环境保护部派出机构人事工作办法（试行）

目录

第一章总则

第二章干部录用与调入

第三章干部任免

第四章民主推荐、民主测评与考察

第五章考核与奖惩

第六章教育与培养

第七章调出、辞职、辞退

第八章工资福利

第九章退休

第十章出国（境）审批

第十一章报告事项

第十二章人事档案管理

第十三章工作纪律

第十四章附则

第一章总则

第一条为认真贯彻执行党的干部路线、方针、政策和国家有关法律、法规，推进派出机构人事工作科学化、民主化、制度化、规范化，建设一支高素质干部队伍，加快推进环境保护历史性转变，建设生态文明，根据《公务员法》、《党政领导干部选拔任用工作条例》（以下简称《干部任用条例》）和有关法律、法规及规章，结合我部实际，制定本办法。

第二条派出机构人事工作以马克思列宁主义、毛泽东思想、邓小平理论和“三个代表”重要思想为指导，深入贯彻落实科学发展观，坚持党管干部原则，坚持德才兼备、以德为先原则，坚持民主、公开、竞争、择优原则，坚持民主集中制原则，坚持依法办事原则。

第三条派出机构参照公务员法及其配套政策法规的规定，对本单位除工勤人员以外的工作人员进行管理，不实行事业单位的专业技术职务、工资、奖金等人事管理制度。

第四条本办法适用于派出机构参照公务员法管理的工作人员管理工作。

第五条派出机构的机构编制管理按照中央和部有关规定执行。

第二章干部录用与调入

第六条派出机构录用担任主任科员以下（含本级，下同）职务工作人员，采用公开考试、严格考察、平等竞争、择优录取的办法。考试录用工作按照中央公务员主管部门和部人事部门统一部署和要求进行，具体程序如下：

（一）制定录用人员计划；

（二）发布人员招考公告；

（三）组织报名与资格审查；

（四）笔试和面试；

（五）按照规定确定考察人选；

（六）组织进行考察和体检；

（七）审批、公示与备案。

第七条新录用工作人员实行一年试用期制度。试用期从正式报到之日起计算，期满经考核合格的，按有关规定及时确定职务和级别；考核不合格的，按有关规定取消录用资格，并报部人事部门和中央公务员主管部门备案。

第八条确定新录用工作人员的职务与级别，由本人对在试用期间的德、能、勤、绩、廉情况进行总结，所在单位对其试用情况进行全面考核，填写《新录用工作人员任职定级审批表》，所在单位领导班子集体研究作出拟任职务和拟定级别的决定，并将任职定级情况抄报部人事部门。

第九条新录用工作人员试用期满，任命职务、确定级别按照下列规定执行：

（一）直接从高等院校毕业生中录用的、没有工作经历的人员：大学本科毕业生、获得双学士学位的大学本科毕业生（含学制为六年以上的大学本科毕业生）、研究生班毕业和未获得硕士学位的研究生，任命为科员，定为二十五级；获得硕士学位的研究生，任命为副主任科员，定为二十四级；获得博士学位的研究生，任命为主任科员，定为二十二级。

（二）其他新录用的工作人员中，具有工作经历的，可根据其资历和工龄，比照所在单位同等条件人员，确定职务与级别。

新录用工作人员任职时间从试用期满之日起计算。

第十条根据工作需要，派出机构从外单位选调干部，必须在规定的编制和职数限额内进行，从严控制，按下列要求办理：

（一）司级干部的选调，由部党组酝酿沟通提出人选，部人事部门组织对人选进行考察并提出意见，报部党组讨论决定。

（二）处级干部的选调，由领导班子集体研究，并征得分管部领导同意后，由部人事部门研究提出意见报分管人事工作部领导审批。

（三）科级以下干部，以参加国家公务员考试录用为主要补充方式。确属工作急需的业务骨干的选调，由领导班子集体研究提出意见，报部人事部门批准。

（四）个人向组织推荐调入人选，必须负责地写出推荐材料并署名。

第十一条派出机构选调干部除应当具备《公务员法》第十一条规定的条件，还应当具备下列资格条件：

（一）司级干部年龄一般在五十五岁以下，处级干部年龄一般在四十五岁以下，科级以下干部年龄一般在三十五岁以下。

（二）具有与拟调岗位要求相当的工作经历和任职资历。具备公务员法及其配套法规规定的晋升至拟任职务累计所需的最低工作年限。

（三）专业技术人员调入派出机构任职的，应当担任副高级专业技术职务二年以上，或者已担任正高级专业技术职务。

（四）在京派出机构从京外选调干部，应当按照有关规定严格控制，原则上只从地市级以上单位或部门选调。

（五）科级以下干部，必须具有公务员身份或参照公务员法管理人员身份，且应当严格控制。

（六）应当具有大学本科以上文化程度，身体健康。

第十二条派出机构选调处级以下干部按照以下程序进行：

（一）领导班子集体研究提出调任人选建议，经批准同意后，征求调出单位意见。

（二）派出机构组织对调任人选进行严格考察，并形成书面考察材料。

考察时，应当听取调任人选所在单位有关领导、群众和干部人事部门、纪检监察机构的意见，并认真查阅干部档案，全面考察了解拟调任人选德、能、勤、绩、廉等方面的表现。应协商调出单位客观、真实地提供反映调任人选现实表现和廉政情况的材料。

（三）根据人选考察情况，领导班子集体讨论决定拟调任人选，并按有关规定在调出、调入单位予以公示。公示期满，对没有反映问题或者反映问题不影响调任的，按照规定进行审批；对反映有严重问题未经查实的，待查实并做出结论后再决定是否调任。

（四）按规定履行审批程序。审批材料包括：请示、干部调任审批表、考察材料、调出单位意见和纪检监察机构提供的廉政情况；按规定需要进行离任审计或者经济责任审计的人员，应当对其进行审计，并提供审计机关的审计结论。

（五）经审批后，办理人员调动手续，并按规定进行参照公务员法管理机构工作人员登记。

第十三条选调的干部调入前是公务员或参照公务员法管理机构工作人员的，一般平级确定职务。从国有企事业单位和社会团体中调入的，按照国家有关规定，根据本人原任职务、工作经历、文化程度、工作需要等因素，参照本单位同等条件干部任职情况确定职务。

从国有企事业单位和社会团体调入担任副处级以上职务的，一般实行任职试用期制，试用期为一年。试用期满考核合格的，正式任职；考核不合格的，另行安排工作。

第十四条拟调入干部在选调考察期间，调出单位突击提拔任职的，不予调入。

第十五条选调干部时应注意本单位工作人员的年龄、专业、素质结构需要和来源分布，避免短时期内从同一单位或地区选调多名干部。

第十六条派出机构考试录用和选调干部实行回避制度。考试录用和选调干部工作中存在应当回避情形的，按照有关规定执行。

第三章干部任免

第十七条派出机构领导班子职数由部党组核定；内设处室处级领导干部职数参照公务员管理的规定由部人事部门核定。

第十八条派出机构领导班子成员职务序列为：主任、副主任。处级领导干部职务序列为：处长、副处长；办公室主任、副主任。

第十九条非领导职务的序列、职数，参照公务员法的规定执行。非领导职务的序列为：巡视员、副巡视员、调研员、副调研员、主任科员、副主任科员、科员、办事员。

第二十条派出机构领导班子成员、巡视员、副巡视员由部党组任免；处级领导职务和非领导职务由所在单位提出意见，报部人事部门审核备案后任免；主任科员以下干部由所在单位任免，任免结果抄报部人事部门。

第二十一条派出机构选拔任用干部必须在规定的职务序列和职数限额内，按照规定程序进行。干部晋升应当具备《公务员法》、《干部任用条例》规定的资格：

（一）晋升正司级、正处级领导职务的，应当分别担任副司级、副处级领导职务两年以上；

（二）晋升副司级、副处级领导职务的，应当分别担任正处级领导职务、主任科员三年以上；

（三）晋升巡视员、副巡视员的，应当分别担任下一级职务五年以上；

（四）晋升调研员、副调研员的，应当分别担任下一级职务四年以上；

（五）晋升主任科员、副主任科员的，应当分别担任下一级职务三年以上。

晋升处级以上领导职务，应当具有五年以上工龄和两年以上基层工作经历，一般应当具有在下一级两个以上职位任职的经历。晋升司级领导职务的，一般还应当有下一级领导职务的任职经历。

第二十二条选拔任用干部，应当逐级进行。表现特别优秀或者工作特殊需要的，可以破格或者越级晋升职务。破格和越级晋升条件和程序按照有关规定执行。

第二十三条提任副处级以上领导职务和由非领导职务改任同级领导职务的，试用期一年。试用期满考核合格的办理正式任职手续，任职时间从试用决定之日起计算。试用期考核不合格，以及在试用期间出现重大失误或犯有严重错误的，按审批权限免去试任职务。免去试任职务的干部，一般按试用前原职级安排适当工作。

第二十四条派出机构科员以上职务的工作人员在定期考核中被确定为不称职的，应予降职；因工作需要或者辞职、退休及其他原因需要免职的，应予免职。

第二十五条实行干部任免职谈话制度。新任或免去派出机构正职的，由部主要领导谈话；新任或免去派出机构副职、巡视员、副巡视员的，由分管部领导或部人事部门谈话；新任或免去处级干部职务的，由派出机构正职或分管副职谈话；新任或免去处级以下干部职务的，由派出机构人事部门谈话。各处（室）主要负责人应与本处室任免职干部谈话。

第二十六条派出机构领导班子集体研究任用干部时，凡在重要问题上有争议的，属于破格提拔的，超过任职年龄需继续留任的，领导班子成员的配偶、子女在本单位提拔的，在作出决定前，应当征求部人事部门的意见。

第四章民主推荐、民主测评与考察

第二十七条派出机构选拔任用司处级干部，严格按照《干部任用条例》和有关法律法规的规定，采取推荐考察或竞争上岗、公开选拔的方式进行。

推荐考察方式的主要程序：酝酿、民主推荐、考察人选公示、民主测评、组织考察、征求意见、讨论决定、任前公示、任职。

竞争上岗和公开选拔，按照《党政机关竞争上岗工作暂行规定》和《公开选拔党政领导干部工作暂行规定》执行。

第二十八条提任司级职务，由部党组酝酿研究；其中，司级领导班子正职由部党组书记与其他部领导酝酿，司级领导班子副职由部党组委托部人事部门负责人沟通听取部领导的意见。提任处级干部，由所在单位领导班子酝酿研究。

司级干部调整补充，由部人事部门根据酝酿方案组织实施。处级干部调整补充，由所在单位人事部门根据酝酿方案组织实施。

第二十九条选拔任用派出机构司处级领导职务和非领导职务，必须经过民主推荐提出考察对象。民主推荐包括会议投票推荐和个别谈话推荐。

民主推荐按照干部管理权限进行。民主推荐司级干部，由部人事部门组织进行；民主推荐处级干部，由派出机构组织进行。

第三十条参加会议投票推荐人员范围为：

推荐司处级干部人选，一般在人选所在派出机构全体干部范围内进行。根据工作需要，推荐派出机构领导班子正职人选，可以在部机关处长以上干部和派出机构、直属单位副司级以上干部范围内进行。

根据工作需要，可以适当扩大会议投票推荐范围。参加会议投票推荐人数不得低于应参加人数的百分之八十。

第三十一条第一次推荐因票数分散，未产生考察人选的，可以差额提出考察对象初步人选名单，进行二次会议投票推荐。参加人员范围一般为人选所在派出机构处级以上干部。根据需要，可以组织考察对象初步人选进行自述。

第三十二条参加个别谈话推荐人员范围为：

推荐派出机构司处级干部人选，一般在人选所在单位司级干部和内设处（室）主要负责人范围内进行。

根据工作需要，可适当扩大个别谈话推荐和征求意见的范围。

第三十三条根据民主推荐结果和有关意见，结合平时考核、年度考核等情况，研究提出考察对象建议人选，并按照干部管理权限确定考察对象。司级干部考察对象由部党组确定，处级干部考察对象由派出机构与部人事部门沟通后集体研究确定。

考察对象人数一般应当多于拟任职务人数，实行差额考察。

第三十四条考察对象应当在一定范围内进行公示，时间为三至七个工作日。

第三十五条组建考察组，采取民主测评、民意测验、个别谈话、查阅干部档案等方式对考察对象进行全面考察。派出机构司级干部的考察由部人事部门负责；处级干部的考察由所在单位负责。

第三十六条民主测评按照德、能、勤、绩、廉五个类别设置测评内容和评价要点，并设置“是否同意其任职”等征求意见栏目。

测评内容主要包括政治态度、大局意识、思想品质，政策水平、组织协调、业务能力，精神状态、工作作风，履行职责成效、解决复杂问题、基础工作，以及廉洁自律等方面情况。

民主测评项目评价意见分为好、较好、较差、差，总体评价意见分为优秀、称职、基本称职、不称职。

第三十七条对考察对象进行民主测评，一般在人选所在派出机构全体干部范围内进行。根据实际情况，可扩大民主测评的范围。拟任职人选，其优秀、称职票应达到三分之二以上，同意任职票应过半数。民主测评基本称职和不称职票达到三分之一或不同意任职票达到二分之一的人员，一般不列为任职人选。

第三十八条个别谈话主要是深入了解领导干部德才素质及表现情况。个别谈话评价内容、评价要点与民主测评相同。个别谈话的参加人员范围为：

（一）考察派出机构正职、巡视员和由副巡视员转任派出机构副职人选，个别谈话在该派出机构司级干部、内设各处（室）主要负责人中进行。

（二）考察派出机构副职、副巡视员和处长人选，个别谈话在该派出机构司级干部、内设处（室）主要负责人及考察对象所在处（室）全体人员中进行。

（三）考察派出机构副处级领导职务、处级非领导职务人选，个别谈话在该派出机构司级干部和考察对象所在处（室）全体人员中进行。

根据实际情况，可扩大个别谈话和征求意见的范围。

第三十九条人事部门按照干部管理权限，就党风廉政等方面情况征求纪检监察部门的意见。对需要进行经济责任审计的，应当委托审计部门按照有关规定进行审计。

第四十条提任派出机构司处级干部的，须在任职前公示七个工作日。司级领导班子成员人选在部机关、各派出机构和直属单位公示；巡视员、副巡视员和处级干部在本单位公示。对公示期间反映的问题按规定进行核查，根据核查结果作出任用、暂缓任用或不予任用的决定。

第五章考核与奖惩

第四十一条按照《党政领导班子和领导干部年度考核办法（试行）》、《公务员考核规定（试行）》的规定，对派出机构工作人员进行考核。考核工作采取平时考核与定期考核相结合的方式进行。

第四十二条派出机构领导班子和班子成员的考核，由部党组负责；其他人员的考核，由派出机构负责，考核结果抄报部人事部门。

第四十三条平时考核主要通过日常干部考察和工作检查、工作总结、听取汇报以及考勤等方式进行。

第四十四条定期考核一般采取年度考核的方式，在每年年末或者翌年年初组织实施，主要程序为：

（一）制定方案、动员部署。制定并公布年度考核工作方案，进行工作动员和部署。

（二）撰写述职述廉报告。根据年度考核内容及有关要求，干部对本年度工作表现进行总结，提交述职述廉报告，并填写《年度考核登记表》。

（三）述职述廉与民主测评。领导班子成员在全体干部会议上述职述廉，部人事部门组织进行民主测评，民主测评在派出机构全体干部范围内进行。其他干部的述职述廉与民主测评由所在单位负责组织。

（四）个别谈话。与派出机构内设处（室）主要负责人以上干部进行个别谈话，听取对派出机构领导班子和班子成员的评价意见。人数较少的单位，可适当扩大人员谈话范围。

（五）确定考核等次。领导班子成员的考核等次由部党组确定。其他干部的考核等次由所在单位主管领导提出建议，报领导班子集体审定。

（六）公示。拟确定为优秀等次的领导班子成员，在部机关、派出机构和直属单位范围内进行公示；其他工作人员，在所在单位进行公示，时间为三至七个工作日。

（七）反馈考核结果。考核情况及结果应及时向考核对象反馈，并由考核对象本人在《年度考核登记表》上签署意见。

对领导班子的年度考核情况，由部人事部门向被考核单位主要负责人或者领导班子集体反馈。对领导干部的年度考核情况，由部人事部门向被考核单位主要负责人或者分别向领导班子成员反馈。

处级以下干部的年度考核情况，由派出机构向干部本人反馈。考核工作结束后，派出机构应将本单位年度考核情况和处级以下干部考核结果报送部人事部门。

（八）《年度考核登记表》存入本人档案，考核结果作为对工作人员进行奖惩、培训、辞退以及调整职务、级别和工资的依据。

第四十五条考核结果分为优秀、称职、基本称职、不称职四个等次。优秀等次人数一般控制在本单位参加考核总人数的百分之十五以内。

第四十六条工作人员中，病、事假累计超过半年的，当年不考核；新录用人员在试用期内参加年度考核，只写评语，不确定等次；挂职锻炼或派出工作人员，在挂职单位或派入单位考核并确定等次，派出未满半年的在原单位考核；派出学习、培训的人员按管理权限考核，根据学习、培训情况确定考核等次。被立案审查或受党纪、政纪处分的，年度考核按有关规定办理。

第四十七条工作人员如对本人考核结果有异议申请复核，应当自知道考核结果之日起三十日内向部人事部门提出书面申请。

无正当理由不参加年度考核的工作人员，经教育后仍然拒绝参加的，可直接确定其考核结果为不称职。

第四十八条对在工作中表现突出、有显著成绩和贡献以及其他突出事迹的工作人员，应当给予奖励。奖励按照《公务员法》、《公务员奖励规定（试行）》和部有关规定执行。

第四十九条派出机构工作人员必须严格遵守纪律。对违反纪律的工作人员视情节轻重分别给予警告、记过、记大过、降级、撤职、开除处分。惩戒按照《公务员法》、《行政机关公务员处分条例》和部有关规定执行。

第六章教育与培养

第五十条干部培养主要包括教育培训、挂职锻炼、交流轮岗等。派出机构应高度重视干部培养工作。

第五十一条教育培训要根据工作需要和职位要求有计划地开展。部人事部门负责总体规划和统筹协调，重点抓好领导班子和人事干部的教育培训工作；各单位负责本单位干部教育培训计划、方案的制定和落实。

第五十二条司处级领导干部每五年应当参加党校、行政学院、干部学院或部人事部门认可的其他培训机构累计不少于三个月的培训，其他干部参加培训的时间一般每年不少于十二天。

第五十三条教育培训以政治理论、政策法规、业务技能等为基本内容，一般采取初任培训、任职培训、业务培训和在职培训等方式。

（一）初任培训是对新录用工作人员进行的培训，一般在试用期内完成，时间不少于十二天。没有参加初任培训或者培训考试、考核不合格的新录用工作人员，不能任职定级。

（二）任职培训是按照新任职务的要求，对晋升司处级领导职务的干部进行的培训，一般应当在任职前或任职后一年内进行，时间原则上不少于三十天。没有参加任职培训或者培训考试、考核不合格的领导干部，应及时进行补训。

（三）业务培训是根据干部从事专项工作的需要进行的专业知识和技能培训。业务培训考试、考核不合格的干部，不得从事专门业务工作。

（四）在职培训是对干部进行的以更新知识、提高工作能力为目的的培训。在职培训考试、考核不合格的干部，年度考核不得确定为优秀等次。

第五十四条派出机构应当建立和完善干部培训档案，对干部参加培训的种类、内容、时间和考试考核结果等情况进行登记。干部必须服从组织调训。干部培训情况作为考核领导班子和干部本人的重要内容，是干部职务调整的重要依据之一。

第五十五条鼓励干部利用业余时间参加学习培训。干部参加在职学历（学位）教育须经本单位批准，学习费用按有关规定执行。干部在职学习取得的学历、学位，须按干部管理权限经人事部门按有关规定确认。

第五十六条派出机构司处级干部挂职锻炼，由部人事部门统筹安排。科级以下干部到基层锻炼，由各单位研究决定。

选派锻炼的干部，由派出和接收单位共同管理，以接收单位管理为主。派出单位对干部锻炼情况跟踪了解和考察，考察结果作为干部使用的依据之一。

第五十七条根据国家有关规定和工作需要加强派出机构干部交流轮岗。领导班子成员在同一单位任职满十年的，一般应当交流任职；处级领导干部在同一职位任职满十年的，一般应当在单位内部交流任职。派出机构应根据工作需要加强其他干部的交流轮岗。

第五十八条干部所在单位和干部本人必须服从和执行组织的交流决定。干部应在接到交流轮岗通知后十五个工作日内到新岗位报到，无正当理由拒不到岗的，予以免职或降职，并视情节轻重按有关规定处理。

第七章调出、辞职、辞退

第五十九条派出机构司级干部调出由部党组审批。处级干部调出由所在单位研究提出意见，征得分管部领导同意后，由部人事部门研究提出意见报分管人事工作部领导审批。科级以下干部调出，由所在单位研究提出意见，报部人事部门审批。干部本人要求调出的，应向所在单位提出书面申请。

第六十条干部因出国定居、自费出国留学、脱产上学或者其他原因要求离开单位，不再担任国家公职的，应当向所在单位提出辞职申请，参照调出程序审批后办理辞职手续。有在涉及国家秘密等特殊职位任职或离开上述职位不满脱密期限、重要公务尚未处理完毕且须由本人继续处理、正在接受审查等情形之一的，不得辞职。

第六十一条新录用的工作人员最低服务年限为五年（含试用期）。未满最低服务年限的，不得自行提出调离或出国自费留学、辞去公职的申请。对擅自离职的，给予开除处分。

第六十二条因干部本人原因不适合继续在单位工作的，按审批权限批准后调出，或劝其辞职。

辞退工作人员，由所在单位在核准事实的基础上，参照调出程序审批。

第六十三条干部有下列情形之一的，予以辞退：

（一）连续两年年度考核被确定为不称职的；

（二）不胜任现职工作，又不接受其他安排的；

（三）因单位调整、撤销、合并或缩减编制员额需要调整工作，本人拒绝组织安排的；

（四）旷工或无正当理由逾期不归连续超过十五天，或一年内累计超过三十天的；

（五）不履行义务，不遵守纪律，经教育仍无转变，不适合继续在机关工作，又不宜给予开除处分的。

第六十四条对有下列情形之一的，不得辞退：

（一）因公致残，被确认丧失或者部分丧失工作能力的；

（二）患病或者负伤，在规定的医疗期内的；

（三）女性工作人员在孕期、产期、哺乳期内的；

（四）法律、法规规定的其他不得辞退的情形。

第六十五条经批准调出、辞职、辞退的干部，应自批准之日起一个月内，按规定交接公务并办理有关手续，交还个人使用的公共财物，必要时应当接受财务审计。无正当理由逾期拒不办理手续的，按开除处理。

第六十六条干部辞职或者被辞退，自批准之月的下月起停发工资，其人事档案按规定及时转至人才流动服务机构。被辞退的干部，按国家有关规定领取辞退费。

第八章工资福利

第六十七条派出机构执行国家规定的工资制度、工资标准和工资政策，执行国家规定的各项津贴、补贴标准。

工作人员正常晋升工资，按国家有关规定办理。新录用、调入、晋升职务、降职的工作人员和军队转业干部等，工资和津补贴按照国家有关规定进行核定。

第六十八条派出机构工资变动按下列审批权限进行：

（一）司级干部和人事部门主要负责人因职务变动，需调整职务工资和级别工资的，由部人事部门审批。

（二）处级以下工作人员因职务变动，需调整职务工资和级别工资的，由派出机构负责审批。

（三）国家统一调整工资时，由部人事部门制定调整工资方案，报分管部领导审批后组织实施。

第六十九条对年度考核称职以上的工作人员，发给一次性奖金，奖金数额按国家规定执行。

第七十条对生活特别困难的工作人员给予一定补助费，标准按国家和部有关规定执行。对援藏、援疆、扶贫和挂职锻炼的工作人员给予一定的生活补助，标准按国家和部有关规定执行。

第七十一条实行年休假制度。工作年限已满一年不满十年的，年休假五天；已满十年不满二十年的，年休假十天；已满二十年的，年休假十五天。当年参加工作的，不享受年休假。各派出机构具体参照我部《机关工作人员带薪年休假制度的实施意见》执行。

第七十二条工作人员夫妻双方两地分居一年以上，又不能在公休假日团聚的，在对方未享受探亲假的情况下，可以享受探望配偶的待遇。工作人员的父母均不是本人工作单位所在地居民且在外省（区、市）工作或生活，不能在公休假日团聚的，可以按规定享受探望父母的待遇。

（一）工作人员探望配偶的，每年给予探亲假一次，假期三十天。未婚工作人员探望父母，原则上每年给予探亲假一次，假期二十天。已婚工作人员探望父母的，每四年给予探亲假一次，假期二十天。根据实际需要，给予路程假。

（二）工作人员探望配偶和父母的往返路费按有关规定报销。

（三）享受探亲假的工作人员，由本人提出申请，参照年休假的程序进行审批备案。

第七十三条符合法定结婚年龄的工作人员结婚可享受婚假，假期三天，晚婚（男满二十五周岁、女满二十三周岁）的增加奖励假七天。

第七十四条女职工生育享受九十天产假；经所在单位批准，可以再增加产假三个月，但减少三年独生子女父母奖励费；晚育（女满24周岁）女职工除享受九十天产假（不含剖腹产假）外，增加奖励假三十天，该奖励假也可由男方享受；剖腹产假根据医院出具的证明办理。

第七十五条工作人员因病休假，需持医生诊断证明，经本单位领导批准，可以休病假。

病假两个月之内，工资照发。病假超过两个月的，从第三个月起按下列标准发给工资：工作年限不满十年的，发给本人工资的百分之九十；工作年限满十年的，工资照发。病假累计超过六个月的，按下列标准发给工资：工作年限不满十年的，发给本人工资的百分之七十；工作年限满十年和十年以上的，发给本人工资的百分之八十。

第七十六条干部的配偶、直系亲属、岳父母、公婆死亡后，可请三天（不含往返路途时间）的丧假。

第七十七条第七十一条至第七十六条所述假期，相互之间可独立享受。除年休假、婚假和丧假外，均包括双休日和法定假日。

第九章退休

第七十八条除国家另有规定外，派出机构工作人员符合下列条件之一的，应当退休：

（一）男年满六十周岁的；

（二）处级以下女干部年满五十五周岁的，司级女干部年满六十周岁的；

（三）完全丧失工作能力的。

第七十九条派出机构工作人员工作年限满三十年，或者距国家规定的退休年龄不足五年、且工作年限满二十年的，可以提前退休。提前退休由本人自愿提出书面申请，按照干部管理权限审批。

第八十条工作人员达到退休年龄的前一个月，所在单位应研究提出办理退休的意见，按照干部管理权限审批后，到龄办理退休手续。

司级干部退休，部人事部门征得分管部领导同意后报分管人事工作部领导审批，分管部领导谈话；处级及处级以下干部退休，部人事部门审批，所在单位正职或分管副职谈话。

第八十一条退休费从批准退休之月的下月起发放，由所在单位按有关规定进行核定，并按照干部管理权限审批。

工作人员退休时，所在单位应填写《退休审批表》，按照干部管理权限签署意见后存入本人档案。

退休人员的福利待遇按国家有关规定执行。

第十章出国（境）审批

第八十二条派出机构因公出国（境）人员的政治审查和因私出国（境）人员审批按照中央和我部有关规定执行。

第八十三条派出机构工作人员因私出国（境），按照事假对待，并应严格按照管理权限进行审批。

（一）派出机构主要负责人因私出国（境），本人提交书面请假报告和有关材料，经部人事部门审核，征求国际司意见并报分管部领导同意后，报部长审批。

（二）派出机构领导班子副职因私出国（境），本人向所在单位提交书面请假报告和有关材料，经所在单位签署意见，由部人事部门审核，征求国际司意见后，报分管部领导审批。

（三）派出机构处级干部因私出国（境），本人向所在单位提交书面请假报告和有关材料，一般由所在单位主要负责人审批。无审批权的单位，须经所在单位签署审核意见，报部人事部门审批。

（四）退休司级干部因私出国（境），本人向部离退休干部管理机构提交有关材料，经离退休干部管理机构审核后，由部人事部门审批。

（五）派出机构其他人员按《中华人民共和国公民出入境管理法》及其《实施细则》以及部因私出国有关规定办理出国（境）手续，经单位批准同意后，到出入境管理部门办理有关手续。

第八十四条经批准因私出国（境）的工作人员，在境外的时间按有关规定执行。出国（境）人员应当及时将本人离境日期报告单位人事部门，逾期不归者，按有关规定处理。

经批准因私出国（境），其费用（包括往返旅费、在境外医疗费等）自理。

第十一章报告事项

第八十五条派出机构应增强信息报送意识，及时、准确、全面地向部人事部门报送以下信息：

（一）本单位领导班子建设、干部人事管理、人事制度改革、领导干部党风廉政等方面的重大情况和重要活动；

（二）本单位领导班子成员的严重病情、伤情等有关情况，在体检中发现的健康方面的重要情况；

（三）按照规定应当报送的其他重要信息。

第八十六条派出机构应指定专人负责信息报送工作。出现以上情况，应当在事后及时上报。对处于发展或处理过程中的难以及时核实的重要事件、重大问题，应当先报送简要情况，然后及时跟踪续报全面情况。

第八十七条派出机构在报送相关信息工作中，应增强保密意识和纪律观念，做好保密工作。凡不宜公开的重要情况，须严格限制知情范围。

第八十八条根据中共中央办公厅《关于党员领导干部报告个人有关事项的规定》，派出机构副处级以上党员干部应当向组织报告下列事项：

（一）本人的婚姻变化情况；

（二）本人持有因私出国（境）证件的情况；

（三）本人因私出国（境）的情况；

（四）子女与外国人、港澳台居民通婚的情况；

（五）配偶、子女出国（境）定居及有关情况；

（六）配偶、共同生活的子女（指同财同居的子女，下同）私人在国（境）外经商办企业的情况；

（七）配偶、共同生活的子女担任外国公司驻华、港澳台公司驻境内分支机构主管人员的情况；

（八）配偶、子女被司法机关追究刑事责任的情况；

（九）本人认为应当报告的其他事项。

第八十九条应当报告的有关事项，党员干部应当在事后三十日内填写《党员领导干部个人有关事项报告表》，向所在单位报告。因特殊原因不能按时报告的，特殊原因消除后应当及时补报，并说明原因。报告人认为有需要请示的事项，可以向所在单位书面请示。所在单位应当认真研究，及时答复报告人。报告人应当按照组织答复意见办理。

所在单位应当于每年二月一日前将上一年度党员领导干部报告有关事项的情况汇总后报部人事部门。部人事部门应当于每年三月一日前将各派出机构上一年度党员领导干部报告有关事项的情况汇总后报部党组和驻部纪检组。

第九十条不按规定报告、不如实报告、隐瞒不报或不按组织答复意见办理的，视情节轻重，采取批评教育、限期改正、责令做出检查、诫勉谈话、通报批评等方式处理。

第九十一条根据中共中央办公厅《关于党政机关县（处）级以上领导干部收入申报的规定》，副处级以上干部应当填写《处以上领导干部收入申报表》。各单位负责接受本单位申报人的收入申报，并及时将领导班子成员的收入申报材料报部人事部门备案。

第十二章人事档案管理

第九十二条派出机构领导班子成员、巡视员、副巡视员、人事部门主要负责人的档案由部人事部门管理；其他工作人员的档案由各单位管理。

第九十三条人事档案信息的归档按下列要求进行：

（一）工作人员接受培训或学历证明等材料原件，按照管理权限由人事部门审核后，归入本人档案；

（二）工作人员的年度考核登记表、考察材料、《干部任免审批表》、工资变动审批（备案）表和奖惩审批表等材料，由所在单位及时归入本人档案；

（三）工作人员婚姻及其配偶、子女学习、工作等情况发生变化，本人应当在三十日内向所在单位人事部门报告，人事部门核实后及时变更干部管理信息。涉及领导班子成员、巡视员、副巡视员、人事部门主要负责人的上述情况变化，所在单位人事部门要及时将变化情况报告部人事部门。

第九十四条管理人事档案，应建立健全并严格执行以下制度：

（一）人事档案材料收集制度。凡是应收入干部人事档案的材料均应按时间、按要求归入个人档案。

（二）人事档案材料鉴别归档制度。要按规定判断材料是否属归档范围，未经鉴别和批准，不得擅自销毁干部档案材料。

（三）人事档案查（借）阅制度。查（借）阅档案，必须经过批准，履行登记手续。任何组织不得批准任何人查（借）阅本人及本人公务回避对象的档案。严禁查（借）阅人在档案材料上涂改、划圈、增删、折叠或损坏档案材料，并不得泄露档案内容。未经同意，不得擅自摘抄、复制档案内容。

（四）人事档案传递制度。人事档案必须通过机要交通或派专人送取，严禁邮寄或交干部本人自带。传递档案要填写传递单，并催要回执。

（五）人事档案保管保密制度。各单位应按有关规定建立可靠的档案保管库房。严禁任何人私自保管他人档案。严格遵守保密规定，防止人事档案毁损、丢失、散落、失密、泄密等事故发生。

第十三章工作纪律

第九十五条各单位要增强纪律观念，严格按照《干部任用条例》规定的原则、条件、资格和程序选拔任用干部，切实加强对干部人事工作的领导，加强监督检查，坚决防止和整治用人上的不正之风，对违反《干部任用条例》的行为，要按照有关规定严肃处理。

第九十六条各单位要严肃干部人事工作纪律，严格对机构编制，干部职级、待遇和领导干部职数的管理，严禁自行设置、变更机构，严禁突破核定编制随意进人，严禁突破现有规定自行提高干部的职级待遇和擅自增加领导职数，严禁随意自设新的领导职务名称。对违反上述规定的，要坚决予以纠正。

第九十七条各单位人事干部要坚持原则，公道正派，坚持讲党性、重品行、作表率，模范遵守党的干部人事工作纪律和各项规定，认真执行组工干部“十严禁”纪律要求，自觉抵制用人上的不正之风。

第十四章附则

第九十八条派出机构人事管理的现行规定与本办法不一致的，以本办法为准；国家对参照公务员法管理机构人事管理工作有新规定的，从其规定。

第九十九条本办法由部行政体制与人事司负责解释。

第一百条本办法自发布之日起施行。

附件三：

环境保护部直属事业单位人事工作办法（试行）

目录

第一章总则

第二章公开招聘

第三章考核

第四章干部任免

第五章民主推荐、民主测评与考察

第六章奖惩

第七章职称管理与干部培养

第八章工资福利

第九章解聘与辞聘

第十章退休

第十一章出国（境）审批

第十二章报告事项

第十三章人事档案管理

第十四章工作纪律

第十五章附则

第一章总则

第一条为认真贯彻执行党的干部和人才路线、方针、政策，推进直属事业单位（简称“直属单位”）人事工作的科学化、民主化、制度化、规范化，深化干部人事制度改革，建设一支高素质干部和人才队伍，加快推进环境保护历史性转变，建设生态文明，根据国家有关法律、法规和政策，结合我部实际，制定本办法。

第二条直属单位人事工作以马克思列宁主义、毛泽东思想、邓小平理论和“三个代表”重要思想为指导，深入贯彻落实科学发展观，坚持党管干部、党管人才原则，坚持德才兼备、以德为先原则，坚持民主、公开、竞争、择优原则，坚持民主集中制原则，坚持依法办事原则。

第三条本办法适用于直属单位和与其建立人事关系的管理人员、专业技术人员和工勤人员。

由部负责管理的社会团体，依照本办法执行。参照公务员法管理的派出机构，另行规定。

第四条直属单位人事工作按照干部管理权限进行，实行部统一领导、分类指导，各单位分级负责的管理体制。

第五条直属单位的基本用人制度是聘用制度，各单位要按照公开招聘、择优聘用、平等自愿、协商一致的原则，与工作人员通过签订聘用合同，明确单位与被聘用人员的责、权、利，保证双方的合法权益。

在直属单位建立和推行岗位管理制度，具体实施办法另行规定。

第六条直属单位的机构编制管理按照中央和部有关规定执行。

第二章公开招聘

第七条直属单位除国家政策性安置、上级调配安排以及确需通过其他方式选拔补充的人员外，在管理人员、专业技术人员和工勤人员岗位上出现空缺，都要按照《事业单位公开招聘人员暂行规定》的要求，面向社会公开招聘。

第八条经费来源部分或全部由财政补助的直属单位，公开招聘工作人员应在编制内或核定岗位内进行。

第九条公开招聘人员按以下程序进行：

（一）制定招聘计划。招聘计划主要包括以下内容：招聘的岗位及条件、招聘的时间、招聘人员的数量、采用的招聘方式等。

（二）发布招聘信息。直属单位应当通过各种渠道广泛发布信息，扩大选人用人范围。招聘信息应当载明直属单位基本简介、招聘的岗位、招聘人员数量及待遇；应聘人员条件；报名方式；考试、考核时间（时限）、内容、范围等事项。

（三）组织报名。受理应聘人员的申请，对资格条件进行审查。

（四）考试。考试内容为招聘岗位所必需的专业知识、业务能力和工作技能。考试一般采取统一笔试、面试的方式进行。

（五）考核。对通过笔试、面试的应聘人员，直属单位组织对其进行思想政治素质、道德品质、业务能力、工作实绩、廉洁自律等情况进行考核，并对应聘人员资格条件进行复核。

（六）体检。对通过考核的人员，应当到指定医院进行体检。

（七）确定拟聘人员。根据考试、考核和体检结果，经直属单位领导班子集体研究择优确定拟聘人员。

（八）公示。对拟聘人员应当在适当范围内进行公示，公示期为七个工作日。

（九）签订聘用合同，办理聘用手续。直属单位法定代表人或者其委托人与受聘人员签订聘用合同，确立人事关系。直属单位公开招聘的人员按照规定实行试用期制度。试用期包括在聘用合同期限内。试用期合格的，正式聘用；不合格的，取消聘用。

第十条因特殊需要，直属单位从外单位选调干部，必须在规定的编制和职数限额内进行，从严控制，按下列要求办理：

（一）司局级干部的选调，由部党组酝酿沟通提出人选，部人事部门组织对人选进行考察并提出意见，报部党组讨论决定。

（二）处级干部的选调，由直属单位领导班子集体研究提出人选，征得分管部领导同意，报经部人事部门批准后按照规定程序办理。

（三）处级以下干部由直属单位领导班子集体研究决定后，按照规定程序选调。

（四）个人向组织推荐调入人选，必须负责地写出推荐材料并署名。

第十一条选调司局级干部年龄一般在五十五岁以下，处级干部年龄一般在四十五岁以下，处级以下干部年龄一般在三十五岁以下。选调干部应当具有与拟调岗位要求相当的工作经历和任职资历；应当具有大学本科以上文化程度，身体健康。

在京直属单位从京外选调干部，应当按照京外调干规定严格控制，原则上只从地市级以上单位或部门选调。

第十二条选调的干部需要任职的，按照干部管理权限进行审批或备案。

拟调入干部在选调考察期间，调出单位突击提拔任职的，不予调入。

第十三条直属单位公开招聘人员、选调干部实行回避制度。凡与单位领导班子成员有夫妻关系、直系血亲关系、三代以内旁系血亲或者近姻亲关系的人员，不得应聘或调入该单位人事、财务、纪检岗位，以及有直接上下级领导关系的岗位。

单位负责人和工作人员在办理人员聘用、干部调入事项时，涉及与本人有上述亲属关系或者其他可能影响人员聘用、干部调入公正的，应当主动申请回避。

第三章考核

第十四条直属单位领导班子和领导班子成员，由部党组负责考核；直属单位其他工作人员，由本单位负责考核。

第十五条考核分为平时考核和定期考核。平时考核随时进行，定期考核采取年度考核方式，对领导班子和领导班子成员还要进行聘任期考核，定期考核以平时考核为基础。

考核结果作为工作人员晋升、聘任、奖惩、培训、解聘、辞退以及调整工资待遇的依据。

第十六条年度考核工作一般在每年年末或者翌年年初进行，按管理权限由人事部门研究提出方案，按程序报批后组织实施。具体程序为：考核准备、述职述廉、民主测评、个别谈话、形成考核材料、评定考核结果、反馈考核结果。

第十七条对领导班子的评价意见分为好、较好、较差、差四个等次。

领导班子成员和工作人员的考核等次分为优秀、称职、基本称职、不称职四个等次。

第十八条评为优秀等次的人员，其民主测评的优秀得票率不得低于二分之一；评为称职等次的人员，其民主测评的优秀和称职得票率不得低于三分之二；民主测评的基本称职和不称职得票率超过三分之一的人员，经组织考核认定后，应当确定为基本称职等次；民主测评的不称职得票率超过三分之一的人员，经组织考核认定后，应当确定为不称职等次。

第十九条考核为好的领导班子及考核为优秀等次的人员，根据有关规定给予相应的表彰和奖励。

对领导干部在年度考核中优秀和称职票达不到三分之二、经组织考核认定为不胜任现职岗位的，应当进行诫勉谈话或工作调整；对不称职票超过三分之一，经组织考核认定为不称职的，应当视具体情况分别作出免职、责令辞职等组织处理。

第二十条直属单位工作人员中，病、事假累计超过半年的，当年不考核；新聘用人员在试用期内参加年度考核，只写评语，不确定等次；挂职锻炼或派出工作人员，在挂职单位或派入单位考核并确定等次，派出未满半年的在本单位考核；派出学习、培训人员按管理权限考核，根据学习、培训情况确定考核等次。被立案审查或受党纪、政纪处分的，年度考核按有关规定办理。

第四章干部任免

第二十一条直属单位司局级领导班子成员和副局级以上干部的任免，由部党组管理；直属单位处级领导班子成员的任免，由部党组委托部人事部门管理。直属单位中层干部（指内设二级机构正副职）的任免，由所在单位党委（不设党委的，领导班子集体）研究决定，征得分管部领导同意后，报部人事部门备案；其中直属单位人事、财务、党务、纪检等部门主要负责人的任免，应事先征求部人事部门和相关部门意见。其他干部的任免，由所在单位领导班子集体研究决定。

第二十二条直属单位选拔任用领导班子成员和中层干部，应符合《党政领导干部选拔任用工作条例》规定的基本条件和任职资格。

（一）应当具有五年以上工龄和两年以上基层工作经历。

（二）由副职提任正职的，应当在副职岗位工作两年以上；由下级正职提任上级副职的，应当在下级正职岗位工作三年以上。

（三）提任领导班子成员的，一般应当具有在下一级两个以上职位任职的经历；由专业技术人员提任副处级领导职务的，一般应当具有中级技术职称五年以上或具有高级技术职称。

（四）一般应当具有大学专科以上文化程度，其中司局级干部一般应当具有大学本科以上文化程度。

（五）应当经过党校、行政院校或者组织（人事）部门认可的其他培训机构五年内累计三个月以上的培训，确因特殊情况在提任前未达到培训要求的，应当在提任后一年内完成培训。

（六）身体健康。

（七）提任党的领导职务的，应当符合《中国共产党章程》规定的党龄要求。

第二十三条直属单位领导班子成员实行聘任制，每任聘期一般为四年，可以连聘连任。

直属单位领导班子成员聘任期间享受国家和部规定的相应政治待遇、工资待遇和生活福利待遇。

第二十四条加大直属单位领导班子成员交流力度。在一个职位任职满两个聘期的，一般应当进行交流轮岗。

第二十五条建立健全直属单位领导班子和领导班子成员任期目标责任制，加强对任期目标的绩效考核，把任期目标考核结果作为对领导班子成员进行任免、奖惩、培训的重要依据。

第二十六条直属单位中层干部实行聘任制。聘任中层干部必须在规定的职数限额内进行，一般应逐级晋升。特别优秀的年轻干部或者工作特殊需要的，可以破格提拔。

第二十七条直属单位聘任中层干部，有下列情况之一的，须在作出任免决定前征求部人事部门的意见：

（一）提拔任用中在重要问题上有争议的；

（二）属于破格提拔的；

（三）超过任职年龄继续留任的；

（四）领导班子成员的配偶、子女在本单位提拔的。

第二十八条新提拔任职的直属单位中层以上领导干部，实行任职试用期制度，试用期为一年。

第二十九条领导干部在任职期间，因健康原因一年以上不能坚持正常工作或离职学习一年以上的应予免职；任期内调离本单位的，从调离之日起职务自然免除。

第三十条实行干部任（免）职谈话制度。新任或免去直属单位司局级领导班子正职的，由部主要领导谈话；新任或免去直属单位司局级领导班子副职的，由分管部领导或部人事部门谈话；新任或免去直属单位处级领导班子正、副职的，由部人事部门谈话；新任或免去中层干部职务的，由直属单位正职或分管副职谈话；新任或免去中层以下干部职务的，由直属单位人事部门谈话。

第五章民主推荐、民主测评与考察

第三十一条直属单位选拔任用领导班子成员和中层干部，采取推荐考察或竞聘上岗、公开选拔的方式进行。

推荐考察方式的主要程序：酝酿、民主推荐、考察对象公示、民主测评、组织考察、征求意见、讨论决定、任前公示、任职。

竞聘上岗和公开选拔，参照《党政机关竞争上岗工作暂行规定》和《公开选拔党政领导干部工作暂行规定》执行。

第三十二条提任司局级职务，由部党组酝酿研究；其中，司局级领导班子正职由部党组书记与其他部领导酝酿，司局级领导班子副职由部党组委托部人事部门负责人沟通听取部领导的意见。提任处级领导班子成员，由部人事部门司务会议酝酿研究并报分管部领导、分管人事工作部领导同意。提任直属单位中层干部，由所在单位领导班子酝酿研究。

第三十三条直属单位领导班子成员调整补充，由部人事部门根据酝酿方案组织实施。直属单位中层干部调整补充，由所在单位人事部门根据酝酿方案组织实施。

第三十四条选拔任用直属单位领导班子成员和中层干部，必须经过民主推荐提出考察对象。民主推荐包括会议投票推荐和个别谈话推荐。

直属单位领导班子成员的民主推荐，由部人事部门组织进行；直属单位中层干部的民主推荐，由直属单位组织进行。

第三十五条参加会议投票推荐人员范围为：

推荐直属单位领导班子成员和中层干部人选，在人选所在单位进行，五十人以下的单位为全体在职人员；五十人以上的单位为中层以上干部。根据工作需要，推荐司局级领导班子正职人选，可以在部机关处长以上干部和派出机构、直属单位副司局级以上干部范围内进行。特殊情况，直属单位与部人事部门沟通后确定参加会议投票推荐人员范围。

根据工作需要，可以适当扩大会议投票推荐范围。参加会议投票推荐人数不得低于应参加人数的百分之八十。

第三十六条第一次推荐因票数分散，未产生考察人选的，可以差额提出考察对象初步人选名单，进行二次会议投票推荐。参加人员范围一般为人选所在单位中层以上干部。根据需要，可以组织考察对象初步人选进行自述。

第三十七条参加个别谈话推荐人员范围为：

推荐直属单位领导班子成员和内设二级机构正职人选，一般在人选所在单位领导班子成员和内设二级机构主要负责人范围内进行。根据工作需要，推荐司局级领导班子正职人选，可以在部机关各部门和派出机构、直属单位主要负责人范围内进行。

推荐直属单位内设二级机构副职人选，一般在人选所在单位领导班子成员、单位综合部门和所在部门主要负责人范围内进行。

根据工作需要，可适当扩大个别谈话推荐范围。

第三十八条根据民主推荐结果和有关意见，结合平时考核、年度考核等情况，研究提出考察对象建议人选，并按照干部管理权限确定考察对象。

司局级干部考察对象由部党组确定，处级领导班子成员考察对象由部人事部门司务会议研究后报分管部领导和分管人事工作部领导同意；直属单位中层干部考察对象由单位领导班子会议研究确定，特殊情况应与部人事部门沟通后确定。

考察对象人数一般应当多于拟任职务人数，实行差额考察。

第三十九条考察对象应当在一定范围内进行公示，时间为三至七个工作日。

第四十条提任直属单位领导班子成员和司局领导职务，由部人事部门组成考察组进行考察；提任直属单位中层干部，由所在单位组成考察组进行考察。

干部考察采取民主测评、民意测验、个别谈话、查阅干部档案等方式对考察对象进行全面考察。

第四十一条民主测评按照德、能、勤、绩、廉五个类别设置测评内容和评价要点。测评内容主要包括政治态度、大局意识、思想品质，政策水平、组织协调、业务能力，精神状态、工作作风，履行职责成效、解决复杂问题、基础工作，以及廉洁自律等方面情况。

直属单位可根据本单位实际情况，适当设置具有本单位或者岗位特点的测评内容。

第四十二条民主测评项目评价意见分为好、较好、较差、差，总体评价意见分为优秀、称职、基本称职、不称职。对拟提拔考察对象，应当设置“是否同意其提拔使用”等征求意见栏目。

第四十三条对考察对象进行民主测评的人员范围，一般与会议投票推荐的人员范围相同。测评对象为中层以下干部的，干部所在二级机构全体在职人员应当参加。

根据实际情况，可扩大民主测评的范围。拟任职人选，其优秀、称职票应达到三分之二以上，同意任职票应过半数。民主测评基本称职和不称职票达到三分之一或不同意任职票达到二分之一的人员，一般不列为任职人选。

第四十四条干部考察个别谈话主要是深入了解领导干部德才素质及表现情况。个别谈话评价内容、评价要点与民主测评相同。干部考察个别谈话的参加人员范围为：

（一）考察直属单位领导班子正职人选，个别谈话在考察对象所在单位领导班子成员、内设二级机构主要负责人中进行。

（二）考察直属单位领导班子副职和内设二级机构正职人选，个别谈话在考察对象所在单位领导班子成员、内设二级机构主要负责人及考察对象所在部门全体人员（十人以上的部门，为具有中级以上专业技术职称人员）中进行。

（三）考察直属单位二级机构副处级领导职务人选，个别谈话在考察对象所在单位领导班子成员、综合处室主要负责人和考察对象所在部门全体人员（十人以上的部门，为具有中级以上专业技术职称人员）中进行。

根据实际情况，可扩大个别谈话和征求意见的范围。

第四十五条与拟提拔考察对象谈话，一般采取考察组集体面谈的方式进行，进一步了解其思想水平、适应职位能力、发展潜力和心理素质等方面的情况，比较对照不同考察环节的评价意见，核实反映的有关问题，深化对考察对象的了解。

第四十六条人事部门按照干部管理权限，就党风廉政等方面情况征求有关部门的意见。对需要进行经济责任审计的，应当委托审计部门按照有关规定进行审计。

第四十七条提任直属单位领导班子成员和中层干部的，须在任职前公示七个工作日。司局级领导班子成员人选在部机关、各派出机构和直属单位公示；处级领导班子成员和中层干部在本单位公示。对公示期间反映的问题按规定进行核查，根据核查结果作出任用、暂缓任用或不予任用的决定。

第六章奖惩

第四十八条对在工作中表现突出、有显著成绩和贡献，或在突发事件中表现突出的集体和个人，应当给予奖励。

对直属单位领导班子和领导班子成员的奖励，一般授予“先进集体”、“先进个人”等荣誉称号。对直属单位中层以下干部职工的奖励，由所在单位研究确定具体办法。

第四十九条奖励审批权限为：直属单位领导班子及其成员的奖励由部党组研究决定；直属单位中层以下干部的奖励由各单位领导班子研究决定。

第五十条奖励审批的程序是：

（一）提出意见。根据工作业绩，在征求群众意见的基础上，集体研究，提出奖励意见或建议。

（二）申报。申报奖励的材料包括：申请奖励报告、《奖励审批表》、事迹材料和其他需要说明的材料。

（三）审批、公示。经人事部门审核后，按照审批权限审批，在一定范围内公示七个工作日。

第五十一条处分的种类为：警告、记过、记大过、降级、撤职、开除六种。行政处分的审批及解除，按照干部管理权限，根据有关规定办理。

第七章职称管理与干部培养

第五十二条直属单位应按照国家有关规定开展专业技术职务评聘工作。

初、中级专业技术职务任职资格的评审工作由各单位评审委员会评审或者委托其他单位相应专业的评审委员会评审。高级专业技术职务任职资格的评审工作由部高级专业技术职务资格评审委员会负责，或者由部人事部门委托相应专业的高级专业技术职务资格评审委员会评审。

第五十三条专业技术职务的聘任，在核定的各单位专业技术职务限额内，在有任职资格的专业技术人员中选聘。初、中级专业技术职务聘期一般不超过三年，高级专业技术职务聘期一般不超过五年，均可以连聘连任。受聘期间享受所聘职务的工资、福利待遇。

第五十四条直属单位工人按照专业技术岗位和普通岗位由各单位进行管理，技术岗位工人的考核与晋升按照国家有关规定进行。

第五十五条各直属单位要着眼于事业长远发展的需要，通过加大干部轮岗交流和培训等方式加强后备干部和年轻干部培养锻炼，为事业发展储备人才。

第五十六条直属单位人员培训实行分级管理。部人事部门负责总体规划、统筹管理和组织协调，并具体负责直属单位领导班子成员和人事干部的教育培训工作，各单位负责本单位干部职工教育培训计划、方案的制定和落实。

第八章工资福利

第五十七条部人事部门按照国家有关规定负责组织制定直属单位工资、津贴、补贴等各项政策，并监督指导各单位组织落实。直属单位应按照国家以及部有关规定，严格执行工资、津贴、补贴等政策。直属单位领导班子成员以及人事部门主要负责人的工资变动，按照程序报部人事部门审批。

第五十八条工作人员工资正常晋升以及因职务变动调整工资，按国家有关工资管理规定执行。新调入人员的工资，根据国家规定的有关具体办法核定。

第五十九条实行年休假制度。工作年限已满一年不满十年的，年休假五天；已满十年不满二十年的，年休假十天；已满二十年的，年休假十五天。当年参加工作的，不享受年休假。直属单位可以根据国家有关法规和部有关规定制定本单位年休假制度实施细则。

第六十条工作人员夫妻双方两地分居一年以上，又不能在公休假日团聚的，在对方未享受探亲假的情况下，可以享受探望配偶的待遇。工作人员的父母均不是本人工作单位所在地居民且在外省（区、市）工作或生活，不能在公休假日团聚的，可以按规定享受探望父母的待遇。

（一） 工作人员探望配偶的，每年给予探亲假一次，假期三十天。未婚工作人员探望父母，原则上每年给予探亲假一次，假期二十天。已婚工作人员探望父母的，每四年给予探亲假一次，假期二十天。根据实际需要，给予路程假。

（二） 工作人员探望配偶和父母的往返路费按有关规定报销。

（三） 享受探亲假的工作人员，由本人提出申请，参照年休假的程序进行审批备案。

第六十一条符合法定结婚年龄的工作人员结婚可享受婚假，假期三天，晚婚（男满二十五周岁、女满二十三周岁）的增加奖励假七天。

第六十二条女职工生育享受九十天产假；经所在单位批准，可以再增加产假三个月，但减少三年独生子女父母奖励费；晚育（女满二十四周岁）女职工除享受九十天产假（不含剖腹产假）外，增加奖励假三十天，该奖励假也可由男方享受；剖腹产假根据医院出具的证明执行。

第六十三条工作人员因病休假，需持医生诊断证明，经本单位领导批准，可以休病假。

病假两个月之内，工资照发。病假超过两个月的，从第三个月起按下列标准发给工资：工作年限不满十年的，发给本人工资的百分之九十；工作年限满十年的，工资照发。病假累计超过六个月的，按下列标准发给工资：工作年限不满十年的，发给本人工资的百分之七十；工作年限满十年和十年以上的，发给本人工资的百分之八十。

第六十四条干部的配偶、直系亲属、岳父母、公婆死亡后，可请三天（不含往返路途时间）的丧假。

第六十五条第五十九条至第六十四条所述假期，相互之间可独立享受。除年休假、婚假和丧假外，均包括双休日和法定假日。

第九章解聘与辞聘

第六十六条直属单位要规范解聘辞聘制度。聘用单位、受聘人员双方经协商一致，可以解除聘用合同。

第六十七条受聘人员有下列情形之一时，直属单位可以随时单方面解除聘用合同：

（一）连续旷工超过十个工作日或者一年内累计旷工超过二十个工作日的；

（二）未经聘用单位同意，擅自出国或者出国逾期不归的；

（三）违反工作规定或者操作规程，发生责任事故，或者失职、渎职，造成严重后果的；

（四）严重扰乱工作秩序，致使聘用单位、其他单位工作不能正常进行的；

（五）被判处拘役、有期徒刑缓刑以及有期徒刑以上刑罚收监执行，或者被劳动教养的。

第六十八条对在试用期内被证明不符合本岗位要求又不同意单位调整其工作岗位的，直属单位也可以随时单方面解除聘用合同。

第六十九条受聘人员有下列情形之一的，直属单位可以单方面解除聘用合同，但是应当提前三十日以书面形式通知拟被解聘的受聘人员：

（一）受聘人员患病或者非因工负伤，医疗期满后，不能从事原工作也不能从事由聘用单位安排的其他工作的；

（二）受聘人员年度考核或者聘期考核不合格，又不同意聘用单位调整其工作岗位的，或者虽同意调整工作岗位，但到新岗位后考核仍不合格的。

第七十条受聘人员有下列情形之一的，直属单位不得解除聘用合同：

（一）受聘人员患病或者负伤，在规定的医疗期内的；

（二）女职工在孕期、产期和哺乳期内的；

（三）因工负伤，治疗终结后经劳动能力鉴定机构鉴定为1至4级丧失劳动能力的；

（四）患职业病以及现有医疗条件下难以治愈的严重疾病或者精神病的；

（五）受聘人员正在接受纪律审查尚未作出结论的；

（六）属于国家规定的不得解除聘用合同的其他情形的。

第七十一条有下列情形之一的，受聘人员可以随时单方面解除聘用合同：

（一）在试用期内的；

（二）考入普通高等院校的；

（三）被录用或者选调到国家机关工作的；

（四）依法服兵役的。

第七十二条除上述情形外，受聘人员提出解除聘用合同未能与聘用单位协商一致的，受聘人员应当坚持正常工作，继续履行聘用合同；六个月后再次提出解除聘用合同仍未能与聘用单位协商一致的，即可单方面解除聘用合同。

第七十三条受聘人员经聘用单位出资培训后解除聘用合同，对培训费用的赔偿在聘用合同中有约定的，按照合同的约定赔偿。受聘人员解除聘用合同后违反规定使用或者允许他人使用原所在聘用单位的知识产权、技术秘密的，依法承担法律责任。涉密岗位受聘人员的解聘或者工作调动，应当遵守国家有关涉密人员管理的规定。

第七十四条有下列解除聘用合同情形之一的，聘用单位应当根据被解聘人员在本单位的实际工作年限向其支付经济补偿：

（一）聘用单位提出解除聘用合同，受聘人员同意解除的；

（二）受聘人员患病或者非因工负伤，医疗期满后，不能从事原工作也不能从事由聘用单位安排的其他工作，聘用单位单方面解除聘用合同的；

（三）受聘人员年度考核不合格或者聘期考核不合格，又不同意聘用单位调整其工作岗位的，或者虽同意调整工作岗位，但到新岗位后考核仍不合格，聘用单位单方面解除聘用合同的。

第七十五条经济补偿以被解聘人员在聘用单位每工作一年，支付其本人一个月的上年月平均工资为标准；月平均工资高于当地月平均工资三倍以上的，按当地月平均工资的三倍计算。聘用单位分立、合并、撤销的，应当妥善安置人员；不能安置受聘人员到相应单位就业而解除聘用合同的，应当按照上述规定给予经济补偿。

第七十六条受聘人员与所在聘用单位的聘用关系解除后，聘用单位要按照国家有关规定及时为职工办理社会保险关系调转手续，做好各项社会保险的衔接工作。

第十章退休

第七十七条除国家另有规定外，直属单位工作人员符合下列条件的，应当退休：

（一）男年满六十周岁的；

（二）女工勤人员年满五十周岁的，处级以下女干部年满五十五周岁的，司级女干部年满六十周岁的；

（三）完全丧失工作能力的。

第七十八条工作人员达到退休年龄的前一个月，所在单位应研究提出办理退休的意见，按照管理权限审批后，到龄办理退休手续。

第七十九条直属单位工作人员退休，按照以下程序办理：

（一）司局级干部退休，部人事部门征得分管部领导同意后报分管人事工作的部领导审批，由分管部领导或者部人事部门谈话。

（二）处级领导班子成员退休，部人事部门征得分管部领导同意后报分管人事工作部领导审批，由部人事部门谈话。

（三）中层干部退休，所在单位领导班子研究并报部人事部门备案，由所在单位分管领导谈话，或者委托人事部门谈话。

（四）其他工作人员退休，所在单位人事部门研究提出意见，征得单位分管领导同意后报分管人事工作领导批准，由人事部门谈话。

第八十条退休费从批准退休之月的下月起发放，由所在单位按有关规定进行核定，并按照管理权限审批。

工作人员退休时，所在单位应填写《退休审批表》，按照管理权限签署意见后存入本人档案。

退休人员的福利待遇按国家有关规定执行。

第十一章出国（境）审批

第八十一条因公出国（境）人员的政治审查和因私出国（境）人员审批按照中央和我部有关规定执行。

第八十二条直属单位工作人员因私出国（境），应严格按照管理权限进行审批。

（一）直属单位主要负责人因私出国（境），本人提交书面请假报告和有关材料，经部人事部门审核，征求国际司意见并报分管部领导同意后，报部长审批。

（二）直属单位副职因私出国（境），本人向所在单位提交书面请假报告和有关材料，经所在单位签署意见，由部人事部门审核，征求国际司意见后，报分管部领导审批。

（三）直属单位处级干部因私出国（境），本人向所在单位提交书面请假报告和有关材料，一般由所在单位主要负责人审批。无审批权的单位，须经所在单位签署审核意见，报部人事部门审批。

（四）退（离）休司局级干部因私出国（境），本人向部离退休干部管理机构提交有关材料，经离退休干部管理机构审核后，由部人事部门审批。

（五）直属单位其他人员按《中华人民共和国公民出入境管理法》及其《实施细则》以及部因私出国有关规定办理出国（境）手续，经单位批准同意后，到出入境管理部门办理有关手续。

第八十三条经批准因私出国（境）的工作人员，在境外的时间按有关规定执行。出国（境）人员应当及时将本人离境日期报告单位人事部门，逾期不归者，按有关规定处理。

经批准因私出国（境），其费用（包括往返旅费、在境外医疗费等）自理。

第十二章报告事项

第八十四条直属单位应增强信息报送意识，及时、准确、全面地向部人事部门报送以下信息：

（一）本单位领导班子建设、干部人事管理、人事制度改革、领导干部党风廉政等方面的重大情况和重要活动；

（二）本单位领导班子成员、院士及知名专家学者的严重病情、伤情等有关情况，在体检中发现的健康方面的重要情况；

（三）按照规定应当报送的其他重要信息。

第八十五条直属单位应指定专人负责信息报送工作，出现以上情况应当及时上报。对处于发展或处理过程中的难以及时核实的重要事件、重大问题，应当先报送简要情况，然后及时跟踪续报全面情况。

第八十六条直属单位在报送相关信息工作中，应增强保密意识和纪律观念，做好保密工作。凡不宜公开的重要情况，须严格限制知情范围。

第八十七条根据中共中央办公厅《关于党员领导干部报告个人有关事项的规定》，直属单位副处级以上党员干部应当向组织报告下列事项：

（一）本人的婚姻变化情况；

（二）本人持有因私出国（境）证件的情况；

（三）本人因私出国（境）的情况；

（四）子女与外国人、港澳台居民通婚的情况；

（五）配偶、子女出国（境）定居及有关情况；

（六）配偶、共同生活的子女（指同财同居的子女，下同）私人在国（境）外经商办企业的情况；

（七）配偶、共同生活的子女担任外国公司驻华、港澳台公司驻境内分支机构主管人员的情况；

（八）配偶、子女被司法机关追究刑事责任的情况；

（九）本人认为应当报告的其他事项。

第八十八条应当报告的有关事项，党员干部应当在事后三十日内填写《党员领导干部个人有关事项报告表》，向所在单位报告。因特殊原因不能按时报告的，特殊原因消除后应当及时补报，并说明原因。报告人认为有需要请示的事项，可以向所在单位书面请示。所在单位应当认真研究，及时答复报告人。报告人应当按照组织答复意见办理。

所在单位应当于每年二月一日前将上一年度党员领导干部报告有关事项的情况汇总后报部人事部门。部人事部门应当于每年三月一日前将各直属单位上一年度党员领导干部报告有关事项的情况汇总后报部党组和驻部纪检组。

第八十九条不按规定报告、不如实报告、隐瞒不报或不按组织答复意见办理的，视情节轻重，采取批评教育、限期改正、责令做出检查、诫勉谈话、通报批评等方式处理。

第九十条根据中共中央办公厅《关于党政机关县（处）级以上领导干部收入申报的规定》，副处级以上干部应当填写《处以上领导干部收入申报表》。各单位负责接受本单位申报人的收入申报，并及时将领导班子成员的收入申报材料报部人事部门备案。

第十三章人事档案管理

第九十一条直属单位领导班子成员、副局级干部、人事部门主要负责人的档案由部人事部门管理；其他工作人员的档案由各单位管理。

第九十二条人事档案信息的归档按下列要求进行：

（一）工作人员接受培训或学历证明等材料原件，按照管理权限由人事部门审核后，归入本人档案；

（二）工作人员的年度考核登记表、考察材料、《干部任免审批表》、工资变动审批（备案）表和奖惩审批表等材料，按照管理权限由人事部门及时归入本人档案；

（三）工作人员婚姻及其配偶、子女学习、工作等情况发生变化的，本人应当在三十日内向所在单位人事部门报告，人事部门核实后及时变更人员管理信息。涉及领导班子成员、司局级干部、人事部门主要负责人的上述情况变化，所在单位人事部门要及时将变化情况报告部人事部门。

第九十三条管理人事档案，应建立健全并严格执行以下制度：

（一）人事档案材料收集制度。凡是应收入干部人事档案的材料均应按时间、按要求归入个人档案。

（二）人事档案材料鉴别归档制度。要按规定判断材料是否属归档范围，未经鉴别和批准，不得擅自销毁干部档案材料。

（三）人事档案查（借）阅制度。查（借）阅档案，必须经过批准，履行登记手续。任何组织不得批准任何人查（借）阅本人及本人公务回避对象的档案。严禁查（借）阅人在档案材料上涂改、划圈、增删、折叠或损坏档案材料，并不得泄露档案内容。未经同意，不得擅自摘抄、复制档案内容。

（四）人事档案传递制度。人事档案必须通过机要交通或派专人送取，严禁邮寄或交干部本人自带。传递档案要填写传递单，并催要回执。

（五）人事档案保管保密制度。各单位应按有关规定建立可靠的档案保管库房。严禁任何人私自保管他人档案。严格遵守保密规定，防止人事档案毁损、丢失、散落、失密、泄密等事故发生。

第十四章工作纪律

第九十四条各单位要增强纪律观念，严格按照《干部任用条例》规定的原则、条件、资格和程序选拔任用干部，切实加强对干部人事工作的领导，加强监督检查，坚决防止和整治用人上的不正之风，对违反《干部任用条例》的行为，要按照有关规定严肃处理。

第九十五条各单位要严肃干部人事工作纪律，严格对机构编制，干部职级、待遇和领导干部职数的管理，严禁自行设置、变更机构，严禁突破核定编制随意进人，严禁突破现有规定自行提高干部的职级待遇和擅自增加领导职数，严禁随意自设新的领导职务名称。对违反上述规定的，要坚决予以纠正。

第九十六条各单位人事干部要坚持原则，公道正派，坚持讲党性、重品行、作表率，模范遵守党的干部人事工作纪律和各项规定，认真执行组工干部“十严禁”纪律要求，自觉抵制用人上的不正之风。

第十五章附则

第九十七条直属单位人事管理的现行规定与本办法不一致的，以本办法为准；国家对直属单位人事管理工作有新规定的，从其规定。

第九十八条直属单位可根据本办法制定本单位人事管理的实施细则。

第九十九条本办法由部行政体制与人事司负责解释。

第一百条本办法自发布之日起施行。
